# Genome sequencing of *Syzygium cumini* (jamun) reveals adaptive evolution in secondary metabolism pathways associated with its medicinal properties

**DOI:** 10.3389/fpls.2023.1260414

**Published:** 2023-11-17

**Authors:** Abhisek Chakraborty, Shruti Mahajan, Manohar S. Bisht, Vineet K. Sharma

**Affiliations:** MetaBioSys Group, Department of Biological Sciences, Indian Institute of Science Education and Research Bhopal, Bhopal, India

**Keywords:** genome sequencing, genome assembly and annotation, adaptive evolution, secondary metabolism, medicinal properties

## Abstract

*Syzygium cumini*, also known as jambolan or jamun, is an evergreen tree widely known for its medicinal properties, fruits, and ornamental value. To understand the genomic and evolutionary basis of its medicinal properties, we sequenced *S. cumini* genome for the first time from the world’s largest tree genus *Syzygium* using Oxford Nanopore and 10x Genomics sequencing technologies. We also sequenced and assembled the transcriptome of *S. cumini* in this study. The tetraploid and highly heterozygous draft genome of *S. cumini* had a total size of 709.9 Mbp with 61,195 coding genes. The phylogenetic position of *S. cumini* was established using a comprehensive genome-wide analysis including species from 18 Eudicot plant orders. The existence of neopolyploidy in *S. cumini* was evident from the higher number of coding genes and expanded gene families resulting from gene duplication events compared to the other two sequenced species from this genus. Comparative evolutionary analyses showed the adaptive evolution of genes involved in the phenylpropanoid-flavonoid (PF) biosynthesis pathway and other secondary metabolites biosynthesis such as terpenoid and alkaloid in *S. cumini*, along with genes involved in stress tolerance mechanisms, which was also supported by leaf transcriptome data generated in this study. The adaptive evolution of secondary metabolism pathways is associated with the wide range of pharmacological properties, specifically the anti-diabetic property, of this species conferred by the bioactive compounds that act as nutraceutical agents in modern medicine.

## Introduction

*Syzygium cumini*, also known as jamun, jambolan, or black plum, is a tropical tree belonging to the Myrtaceae plant family. It is native to the Indian subcontinent and South-East Asia, and is known for its wide range of medicinal properties and typical purple-black berries (Srivastava and Chandra, 2013). *Syzygium*, the clove genus, is the world’s largest tree genus with 1,193 recognized species. They occupy various habitats, medium to large-sized, typically sub-canopy trees, and thus affect the ecosystems of a wide range of organisms. Some of the other species from *Syzygium* genus are - *S. caryophyllatum*, *S. aromaticum*, *S. aqueum, S. grande*, S. *myrtifolium*, etc., which are used as spices or fruits in pharmacology and horticulture industry (Low et al., 2022).

*S. cumini* is an evergreen tree with 30 meters of height, 3.6 meters of girth, and up to 15 meters of bole, and can live more than 100 years (Dagadkhair, 2017). This *Syzygium* species is widely cultivated in tropical countries for its edible fruit (“Jamun”), which has significant economic importance (Madani et al., 2021). *S. cumini* was introduced in several tropical and sub-tropical regions of the world for its commercial applications, such as Southern Africa, West Indies, California, and Israel (Chaudhary and Mukhopadhyay, 2012). The purple-black colored fruits of *S. cumini* are rich in anthocyanin, polyphenol, and tannin content and possess high nutrient values and medicinal properties (Ghosh et al., 2017). Besides this, other parts of the tree, such as wood, leaf, flower, seed, and bark also have various economic and medicinal properties (Chaudhary and Mukhopadhyay, 2012).

All the plant parts of *S. cumini* have therapeutic properties, which are used in various treatments since the Ayurvedic era (Dagadkhair, 2017). The extracts from seed, bark, fruit, leaf, and flower of this species contain various phytochemicals such as glucoside jambolin, flavonoids including anthocyanin, terpenoids, and alkaloids (e.g., jambosine), which confer medicinal properties including anti-allergic, anti-oxidant, anti-diarrhoeal, anti-microbial, anti-inflammatory, anti-cancer, and others (Chaudhary and Mukhopadhyay, 2012; Srivastava and Chandra, 2013; Dagadkhair, 2017; Kumar et al., 2023). Specifically, the fruit seed extracts of *S. cumini* have well-known anti-diabetic properties conferred by the glucoside jambolin present in the seeds (Chaudhary and Mukhopadhyay, 2012; Dagadkhair, 2017). Besides, the leaves and bark extracts of *S. cumini* also have anti-diabetic potential due to the presence of phyenylpropanoids (Srivastava and Chandra, 2013; Correia et al., 2023). Further, *S. cumini* fruits also possess anti-hyperlipidemic, hepatoprotective, anti-ulcer, anti-arthritic, anti-fertility, and anti-pyretic activities (Srivastava and Chandra, 2013). Different parts of this tree are also rich in compounds containing isoquercetin, myrecetin, kaemferol, and others (Ayyanar and Subash-Babu, 2012).

*Syzygium*, the tree genus with the highest number of species, is characterized by rapid speciation events, which resulted in a wide range of ecological and morphological diversity within the genus. A previous study has indicated that an ancient pan-Myrtales Whole Genome Duplication (WGD) event might have contributed to the early stages of diversification in the Myrtales plant order (Low et al., 2022). However, whole genome sequencing of only two *Syzygium* species has been performed until now (Low et al., 2022; Ouadi et al., 2022), and the whole genome and transcriptome assembly of *S. cumini* was not available.

Therefore, in this study, the genome sequencing of *S. cumini* was performed using 10x Genomics linked reads and Oxford Nanopore long reads to assemble its nuclear genome (710 Mbp) and chloroplast genome (158 Kbp). We also report the transcriptome assembly of *S. cumini* for the first time in this study. We inferred *S. cumini* genome to be tetraploid in this study, whereas previous studies have also shown the existence of intraspecific polyploidy (ranging from 2x to 6x) in *S. cumini* species (Ohri, 2015; Sharma et al., 2020). The phylogenetic position of *S. cumini* was resolved with respect to 17 other Eudicot orders, and comparative evolutionary analyses showed the key plant secondary metabolism pathways, such as the phenylpropanoid-flavonoid (PF) biosynthesis pathway, were adaptively evolved in this *Syzygium* species, which are responsible for the immense medicinal properties of this tree.

## Materials and methods

### Genome and transcriptome sequencing

The clean leaves of *S. cumini* were used for DNA-RNA extraction and species identification (**Supplementary Figure 1**). DNA extraction was performed using Carlson lysis buffer (**Supplementary Text**). Quality check and quantification of the extracted DNA were carried out using Nanodrop 8000 spectrophotometer and Qubit 2.0 fluorometer, respectively. Species identification was performed using amplification and Sanger sequencing of the two marker genes - *ITS2* (Internal Transcribed Spacer) and *MatK* (Maturase K), followed by BLASTN of the gene sequences against NCBI non-redundant (nt) database (**Supplementary Text**). The extracted DNA was used to prepare the 10x Genomics library on the Chromium controller instrument using Chromium Genome Library and Gel Bead Kit (10x Genomics), and sequenced on Illumina NovaSeq 6000 instrument. Further, the DNA was purified using Ampure XP magnetic beads (Beckman Coulter, USA), which was used to prepare the Nanopore library with SQK-LSK109 and SQK-LSK110 library preparation kit (Oxford Nanopore Technologies, UK) for sequencing on a MinION Mk1C sequencer.

The RNA was extracted following a similar method that was used for *Syzygium longifolium* species with a few modifications (**Supplementary Text**) (Soewarto et al., 2019). Extracted RNA was washed and purified using a RNeasy mini kit (Qiagen, CA, USA). The RNA quality was diluted ten times and Qubit 2.0 fluorometer was used for quantification using a qubit ss RNA HS kit (Life Technologies, United States). Quality of the RNA was evaluated using High Sensitivity D1000 ScreenTape on Agilent 2200 TapeStation (Agilent, Santa Clara, CA). The RNA library was prepared using TruSeq Stranded Total RNA Library Preparation kit with the Ribo-Zero Plant workflow (Illumina Inc., CA, USA). The transcriptome library was sequenced on Illumina NovaSeq 6000 instrument to generate 150 bp paired-end reads.

### Genome assembly

Genomic characteristics such as genome size, genomic ploidy, and heterozygosity content were predicted using the 10x Genomics short reads before genome assembly. For this, barcode sequences were filtered using proc10xG (https://github.com/ucdavis-bioinformatics/proc10xG). The barcode-filtered reads were further processed using Trimmomatic v0.39 with the filtering parameters - ‘‘TRAILING:20 LEADING:20 MINLEN:60 SLIDINGWINDOW:4:20” (Bolger et al., 2014). The filtered short reads were used to estimate the ploidy level of *S. cumini* genome using Smudgeplot v0.2.2 (Ranallo-Benavidez et al., 2020). Further, these pre-processed reads were used to construct the k-mer count-based histogram with Jellyfish v2.2.10 (Marçais and Kingsford, 2011), which was used to predict the genome size and heterozygosity content with GenomeScope v2 (Ranallo-Benavidez et al., 2020).

Oxford Nanopore long reads were processed to remove adapter sequences using Porechop v0.2.4, which were further error-corrected and *de novo* assembled using Canu v2.2 (Koren et al., 2017). The resultant genome assembly was polished thrice with Pilon v1.23 using the pre-processed 10x Genomics short reads to fix any assembly errors (Walker et al., 2014). Scaffolding was performed with the quality-filtered RNA-Seq reads (filtered using Trimmomatic v0.39), barcode-processed 10x Genomics reads (processed using Longranger basic v2.2.0), and error-corrected Nanopore reads using AGOUTI v0.3, ARCS v1.1.2, and LINKS v1.8.6, respectively (Warren et al., 2015; Zhang et al., 2016; Yeo et al., 2018). Gap-closing of this scaffolded assembly was carried out using the error-corrected Nanopore long reads using LR_Gapcloser (three iterations) (Xu et al., 2018). Finally, the pre-processed 10x Genomics reads were used to again polish the assembly using Pilon v1.23 (Walker et al., 2014), and scaffolds with lengths of ≥5 Kb were retained to construct the final genome assembly of *S. cumini*.

After constructing the final genome assembly, the genomic ploidy level was further verified using nQuire (Weib et al., 2018). The pre-processed linked reads were mapped onto the assembled genome using BWA-MEM v0.7.17 (Li, 2013), and using these alignments, base frequencies were modeled using a Gaussian Mixture Model in nQuire. Log-likelihood values were estimated for each fixed model using the denoised base frequency distribution. The fixed model with the lowest *Δ*log-likelihood value compared to the free model was considered as the predicted ploidy level.

To assess the genome assembly quality, the pre-processed 10x Genomics linked reads, error-corrected Nanopore reads and quality-filtered RNA-Seq reads were mapped onto the assembled genome to calculate the read mapping percentage using BWA-MEM v0.7.17 (Li, 2013), Minimap v2.17 (Li, 2018), and HISAT v2.2.1 (Kim et al., 2015), respectively. BUSCO v5.4.4 was used to check the presence of single-copy orthologous genes in the final genome assembly with embryophyta_odb10 dataset (Simão et al., 2015). Further, LTR Assembly Index (LAI) score was also calculated to evaluate the genome assembly quality using GenomeTools v1.6.1 and LTR_retriever v2.9.0 (Gremme et al., 2013; Ou and Jiang, 2018).

To identify sequence variation in the *S. cumini* genome assembly, the pre-processed linked reads were mapped using BWA-MEM followed by variant calling using BCFtools “mpileup” v1.9 with the parameters - depth ≥30, variant sites quality ≥30, and mapping quality ≥50 (Li, 2013; Danecek et al., 2021).

### Chloroplast genome assembly

The chloroplast genome of *S. cumini* species was assembled with the pre-processed 10x Genomics data using GetOrganelle v1.7.7.0 with embplant_pt as seed database (Jin et al., 2020). The chloroplast genome was annotated using GeSeq in CHLOROBOX with chloroplast genomes of other *Syzygium* species (*S. aromaticum*, *S. forrestii*, *S. jambos*, and *S. malaccense*) available in NCBI RefSeq database as reference sequences (Tillich et al., 2017; Greiner et al., 2019).

Single nucleotide variant (SNV) analysis was performed using the chloroplast genome constructed in this study with three previously reported *S. cumini* chloroplast genomes with the NCBI accessions - GQ870669.3 (Asif et al., 2013), NC_053327.1, and MN095412.1. The chloroplast genomes were aligned using MAFFT v7.310 (Katoh and Standley, 2013), and the alignments were used to identify the single nucleotide variants using DnaSP v6 with a sliding window of 600 bp and a step size of 200 bp (Rozas et al., 2017).

### Genome annotation

The whole genome assembly of *S. cumini* was used for constructing a *de novo* repeat library with RepeatModeler v2.0.3 (Flynn et al., 2020), which was used to soft-mask the *S. cumini* genome with RepeatMasker v4.1.2 (http://www.repeatmasker.org). The repeat-masked genome was used to identify the coding genes with MAKER v3.01.04 genome annotation pipeline using AUGUSTUS as the *ab initio* gene predictor (Stanke et al., 2006; Campbell et al., 2014). For evidence-based alignments, *de novo* transcriptome assembly of *S. cumini* constructed in this study using Trinity v2.14.0 (Haas et al., 2013), and protein sequences of the two sequenced species from *Syzygium* genus - *S. aromaticum* and *S. grande* (Low et al., 2022; Ouadi et al., 2022) and other species from Myrtales order (*Eucalyptus grandis* and *Corymbia citriodora*) available in Ensembl plants release 56 were used (Bolser et al., 2016). A high-confidence coding gene set was constructed with the filtering criteria of AED value <0.5 and coding gene length ≥150 nucleotides.

The completeness of this coding gene set was evaluated using BUSCO v5.4.4 with embryophyta_odb10 database (Simão et al., 2015). Gene expression values (TPM) were also estimated by mapping the quality-filtered RNA-Seq data of *S. cumini* onto the coding gene set (nucleotides) using Kallisto v0.48.0 (Bray et al., 2016).

The genome assembly of *S. cumini* was used for prediction of non-coding RNAs. *de novo* prediction of rRNA and tRNA was performed using Barrnap v0.9 (https://github.com/tseemann/barrnap) and tRNAscan-SE v2.0.9 (Chan et al., 2021), respectively. miRNA sequences were identified using BLASTN (sequence identity 80% and e-value 10^-9^) against the miRBase database (Griffiths-Jones et al., 2008).

### Collinearity analysis

MCScanX was used to analyze the intra-species collinearity for *S. cumini* species using the BLASTP homology alignments of coding genes and GFF annotations (Wang et al., 2012). Further, inter-species collinear blocks were identified between *S. cumini* and *S. grande*, *S. grande* and *S. aromaticum*, and *S. aromaticum* and *S. cumini* using previously available data (Low et al., 2022; Ouadi et al., 2022). Gene duplication analysis was also performed for the three *Syzygium* species using MCScanX.

### Phylogenetic analysis

For constructing the species phylogenetic tree, one Eudicot species from each available plant order (except Myrtales) in Ensembl plants release 56 was considered (Bolser et al., 2016). For Myrtales order, both the species available in Ensembl plants release 56 – *E. grandis* and *C. citriodora* were considered, along with *S. grande* and *S. aromaticum* from previous studies (Low et al., 2022; Ouadi et al., 2022). The selected species from other 17 Eudicot plant orders were – *Arabidopsis thaliana* (Brassicales), *Actinidia chinensis* (Ericales), *Beta vulgaris* (Caryophyllales), *Citrus clementina* (Sapindales), *Cucumis sativus* (Cucurbitales), *Coffea canephora* (Gentianales), *Cynara cardunculus* (Asterales), *Daucus carota* (Apiales), *Gossypium raimondii* (Malvales), *Juglans regia* (Fagales), *Kalanchoe fedtschenkoi* (Saxifragales), *Rosa chinensis* (Rosales), *Populus trichocarpa* (Malpighiales), *Sesamum indicum* (Lamiales), *Solanum tuberosum* (Solanales), *Vigna radiata* (Fabales), and *Vitis vinifera* (Vitales). Alongside, *Zea mays* was considered as an outgroup species.

Proteome files of these 23 species with the longest isoforms for each protein were used for orthogroups construction with OrthoFinder v2.5.4 (Emms and Kelly, 2019). The orthogroups were filtered to extract the fuzzy one-to-one orthogroups using KinFin v1.1 (Laetsch and Blaxter, 2017). Only those orthogroups comprising sequences from all 23 species were considered, and each orthogroup was processed to include only one longest sequence per species. The resultant orthogroups were individually aligned using MAFFT v7.310 (Katoh and Standley, 2013), and filtered and concatenated with BeforePhylo v0.9.0 (https://github.com/qiyunzhu/BeforePhylo). The species phylogenetic tree was constructed with this concatenated alignment using maximum likelihood-based RAxML v8.2.12 with 100 bootstrap values and “PROTGAMMAAUTO” model (Stamatakis, 2014).

### Analysis of gene family evolution

Proteome files of 23 species with the longest isoforms for each protein were used to analyze the expansion/contraction of gene families with CAFÉ v5 (Mendes et al., 2020). Homology-based search results obtained from All-versus-All BLASTP with the protein sequences of all 23 species were clustered using MCL v14.137 (Van Dongen and Abreu-Goodger, 2012). Gene families containing genes from <2 species of the specified clades and ≥100 gene copies for ≥1 species were filtered out as per the suggestions for performing CAFÉ analysis. An ultrametric species phylogenetic tree across the 23 species was constructed using the calibration point for *S. cumini* and *B. vulgaris* (118 years), as reported in the TimeTree database v5 (Kumar et al., 2022). The ultrametric species tree and the filtered gene families were used in the two-lambda (λ) model implemented in CAFÉ v5, where species from Myrtales order were indicated separate λ-value compared to the other species.

### Identification of secondary metabolite biosynthesis-related gene families and biosynthetic gene clusters

For identification of secondary metabolism-related gene families, the protein sequences of the genes involved in secondary metabolite biosynthesis pathways were downloaded from UniProt or KEGG databases for *Arabidopsis thaliana* or other closely related Eudicot species (Kanehisa, 2002; Bateman et al., 2023). The protein sequences were mapped against the *S. cumini* protein sequences using BLASTP with e-value 10^-5^, and the annotations were further verified from the assigned KO (Kegg Orthology) IDs. These secondary metabolite biosynthesis genes of *S. cumini* were identified in the filtered gene families used for CAFÉ analysis, and these families were further analyzed for gene family expansion/contraction. Biosynthetic gene clusters (BGCs) were identified in *S. cumini* genome using plantiSMASH v1.0 with CD-HIT filtering cut-off 0.5 and “–inclusive”, “–borderpredict”, “–all-orfs”, and “–smcogs” parameters (Kautsar et al., 2017).

### Identification of evolutionary signatures in *S. cumini* genes

Comparative analysis was performed to identify evolutionary signatures in *S. cumini* genes across 13 Eudicot species including *S. cumini*. Four other species from Myrtales order itself (*S. grande*, *S. aromaticum*, *E. grandis*, and *C. citriodora*), and species from its closer plant orders were considered for the analysis. Species from other plant orders used in these analyses were – *V. radiata* (order Fabales), *C. sativus* (order Cucurbitales), *J. regia* (order Fagales), *R. chinensis* (order Rosales), *P. trichocarpa* (order Malpighiales), *A. thaliana* (order Brassicales), *G. raimondii* (order Malvales), and *C. clementina* (order Sapindales).

#### Unique amino acid substitution with functional impact

Protein sequences of the 13 species were used for orthogroups construction with OrthoFinder v2.5.4 (Emms and Kelly, 2019). Orthogroups comprising sequences from the 13 species were extracted, and each orthogroup was filtered to retain the longest sequence for each species. The resultant orthogroups were individually aligned with MAFFT v7.310, and from these multiple sequence alignments *S. cumini* genes were identified that showed different amino acids in positions where the other species had the same amino acid. In this analysis, gaps in the alignments and ten positions around the gaps were not considered. Further, impact of the unique substitutions on the protein function was predicted with SIFT using UniProt as a reference database (Ng and Henikoff, 2003).

#### Higher nucleotide divergence

The protein sequence alignments for the orthogroups obtained in the previous step were used for orthogroup-specific phylogenetic tree construction with RAxML v8.2.12 using 100 bootstrap values and “PROTGAMMAAUTO” model (Stamatakis, 2014). Using the gene phylogenetic trees, *S. cumini* genes showing greater branch length values compared to the genes from other species were identified using “adephylo” package in R, and were termed as the genes with higher nucleotide divergence (Jombart and Dray, 2010).

#### Positive selection

The orthogroups constructed across 13 species (nucleotide sequences) were individually aligned with MAFFT v7.310 (Katoh and Standley, 2013). The resultant multiple sequence alignments and the species tree of 13 species (constructed with RAxML) were used to detect the positively selected genes in *S. cumini* with a branch-site model in “codeml” of PAML v4.10.6 (Yang, 2007). Likelihood-ratio test (LRT) and chi-square analysis were performed, and genes qualifying against the null model with FDR-corrected p-values of < 0.05 were identified as the genes showing positive selection in *S. cumini*. Further, Bayes Empirical Bayes analysis was performed to detect the genes with codon sites under positive selection (with >95% probability) for the foreground lineage.

*S. cumini* genes with more than one of the evolutionary signatures – unique substitution with functional impact, positive selection, and higher nucleotide divergence were termed as the genes showing multiple signatures of adaptive evolution (MSA) (Agaba et al., 2016; Jaiswal et al., 2021).

### Functional annotation

*S. cumini* coding gene set was annotated against publicly available databases - Swiss-Prot database using BLASTP (e-value 10^-5^), NCBI-nr database using BLASTP (e-value 10^-5^), and Pfam-A database using HMMER v3.1 (e-value 10^-5^) (Bairoch and Apweiler, 2000; Bateman, 2004; Finn et al., 2011). *S. cumini* coding gene set, including the expanded gene families and the genes with evolutionary signatures, were annotated using eggNOG-mapper v2.1.9 and KAAS v2.1 genome annotation servers (Moriya et al., 2007; Huerta-Cepas et al., 2017). Over-representation analysis using WebGestalt web server was performed to assign Gene Ontology (GO) categories to the MSA genes of *S. cumini* (Liao et al., 2019).

### Gene structure analysis

The key genes associated with phenylpropanoid-flavonoid (PF) biosynthesis pathway and terpenoid biosynthesis pathway were identified in *S. cumini* genome from the functional annotation of the coding genes. Gene families were identified from the CAFÉ analysis, and the longest gene for each gene family was extracted. The genes were mapped separately onto *S. cumini* genome constructed in this study and the previously available *S. aromaticum* (Ouadi et al., 2022) and *S. grande* (Low et al., 2022) genomes using Exonerate v2.4.0 (https://github.com/nathanweeks/exonerate) to construct the exon-intron structures (Chakraborty et al., 2023), and for a comparative analysis across the three *Syzygium* species.

### Identification of plant disease susceptible genes in *S. cumini* genome

To identify the disease susceptible genes (S-genes) in *S. cumini* genome, the coding genes were mapped against the DSP (Disease Susceptibility Genes in Plants) database consisting of 448 S-genes using BLASTN with query coverage 80%, sequence identity 80%, and e-value of 10^-9^ (Kaur et al., 2023).

## Results

### Genome assembly

Species identification was performed using *matK* and *ITS2* marker gene sequencing, which showed 99.65% and 99.7% sequence similarity (the best hits), respectively, with *S. cumini* gene sequences available in NCBI non-redundant nucleotide (nt) database. 120.7 Gb of 10x Genomics data and 14.4 Gb Oxford Nanopore data (read N50 = 10.9 Kb) were generated for genome assembly. Based on the predicted genome size of 730.3 Mbp (using GenomeScope), the genomic data corresponded to 165.3x and 19.7x sequencing coverage for 10x Genomics and Nanopore reads, respectively (**Supplementary Table 1**). Additionally, 15.1 Gb RNA-Seq data was also sequenced from the *S. cumini* leaf tissue.

*S. cumini* genome contained 3.25% heterozygosity (**Supplementary Figure 2**) and was inferred as a tetraploid genome since the distribution of base frequencies at the variable sites showed the smallest Δlog-likelihood value for the tetraploid fixed model (**Supplementary Figure 3A**). The heterozygous k-mer pair distribution showed that 87% of the k-mers represented the total coverage of k-mer pair 4n (**Supplementary Figure 3B**) (Ranallo-Benavidez et al., 2020).

*S. cumini* genome assembly constructed using Canu (Koren et al., 2017) had a size of 706.9 Mbp with 9,704 contigs, N50 value of 102.1 Kb, and 95.4% BUSCO completeness. However, after three rounds of assembly polishing, the BUSCO completeness was improved to 98.1%. After genome assembly scaffolding and other post-processing steps, the final genome assembly had a size of 709.9 Mbp containing 7,702 scaffolds with an N50 value of 179.2 Kb, and the largest scaffold size of 1.6 Mb. The improvement in the genome assembly statistics after each assembly process step is mentioned in **Supplementary Table 2**.

*S. cumini* genome showed the presence of 98.3% complete BUSCOs (64.6% complete and single-copy, and 33.7% complete and duplicated) (**Supplementary Table 3**). The genome assembly also had an LAI (LTR Assembly Index) score of 11.69. Further, 97.83% of barcode-filtered 10x Genomics reads, 93.45% error-corrected Nanopore reads, and 95.25% quality-filtered RNA-Seq reads were mapped onto the genome assembly. A total of 6,184,849 base positions (0.87%) in the genome assembly had sequence variations.

### Chloroplast genome assembly

The chloroplast genome assembly of *S. cumini* showed a circular genome of 158,509 bases with 83 protein-coding genes (**Supplementary Figure 4**). The assembled genome size was comparable to the *S. malaccense* chloroplast genome (Tao et al., 2020). However, the previously reported *S. cumini* chloroplast genome size (Asif et al., 2013) was larger compared to this study, and also larger than the other two *S. cumini* chloroplast genomes available at NCBI database (NCBI accessions: NC_053327.1 and MN095412.1) (**Supplementary Table 4**). The comparative statistics of the sizes of Large Single Copy (LSC), Small Single Copy (SSC), and Inverted Repeat (IR) regions of *S. cumini* chloroplast genomes in this study and the previous study (Asif et al., 2013) and the chloroplast genome of *S. malaccense* (Tao et al., 2020) are mentioned in **Supplementary Table 4**. The LSC region in the *S. cumini* chloroplast genome provided by Asif et al., 2013 was larger compared to the other genomes available at NCBI database, the genome constructed in this study, and the *S. malaccense* chloroplast genome (Tao et al., 2020).

SNV analysis between the *S. cumini* chloroplast genome sequences constructed in this study and the previous report (Asif et al., 2013) showed the presence of 978 positions with nucleotide variations and a nucleotide diversity (π) value of 0.00620. LSC (π = 0.00837) and SSC (π = 0.00843) regions showed higher intraspecies variability compared to the IR-a (π = 0.00169) and IR-b (π = 0.00169) regions, similar to other studies (Silva et al., 2019). However, the SNV analysis of *S. cumini* chloroplast genome sequence constructed in this study with two other *S. cumini* chloroplast genomes available at NCBI database revealed the presence of a lower number of single nucleotide variants - 282 SNVs with π = 0.00179 (NCBI accession - NC_053327.1) and 197 SNVs with π = 0.00125 (NCBI accession - MN095412.1) (**Supplementary Figure 5**).

*S. cumini* chloroplast genome constructed in this study had 83 protein-coding genes, same as the previous study (Asif et al., 2013). Additionally, three pseudogenes were present in *S. cumini* chloroplast genome (Asif et al., 2013), among which *ycf15* could not be annotated in our study using GeSeq in CHLOROBOX tool (Tillich et al., 2017). However, we could map the *ycf15* gene sequence of *S. cumini* (Asif et al., 2013) in the chloroplast genome of this study at the same genomic location with 100% identity and 100% query coverage using BLASTN.

### Genome annotation

A *de novo* repeat library consisting of 2,521 sequences for *S. cumini* genome was used to repeat mask 51.51% of the genome assembly. Among the repeat classes, 49.31% were interspersed repeats, including 8.09% Gypsy and 5.37% Copia elements (**Supplementary Table 5**). Using the repeat-masked genome assembly, coding genes were predicted in *S. cumini* genome using the MAKER pipeline (Campbell et al., 2014).

A total of 204,525 transcripts were assembled with an N50 value of 2,313 bp (**Supplementary Table 6**), which were used as empirical evidence along with the protein sequences of species from Myrtales order during coding genes prediction. 74,657 coding genes were predicted, among which 62,971 were retained (84.35%) after AED-based filtering. Further, length-based filtering was performed to retain 61,195 coding genes in the final high-confidence gene set with an average CDS length of 1,106.2 bp. 32,888 of the coding genes (53.74%) showed relatively higher gene expression (TPM values > 1). Distribution of the coding genes in various KEGG pathways and COG categories are mentioned in **Supplementary Tables 7, 8**.

This coding gene set showed the presence of 92.8% BUSCOs (78.7% complete and 14.1% fragmented) (**Supplementary Table 3**). 92.38% of the genes were annotated using any of the three publicly available databases – NCBI-nr, Pfam-A, and Swiss-Prot (**Supplementary Table 9**). 1,174 tRNAs decoding standard amino acids, 702 rRNAs, and 176 miRNAs were also identified in the assembled genome of *S. cumini*. Further, seven disease susceptible genes (S-genes) were identified in the *S. cumini* coding gene set (**Supplementary Table 10**).

### Collinearity and orthologous gene clustering

Synteny analysis showed the presence of 16.76% intra-species collinearity in *S. cumini* genome. Further, 90.55% of the coding genes were originated by duplication events, whereas, gene duplication analysis using previously available data of *Syzygium* species showed a lesser percentage of duplicated genes in *S. grande* (75.94%) and *S. aromaticum* (85.06%) (Low et al., 2022; Ouadi et al., 2022). Inter-species collinearity analysis showed a higher percentage of collinear genes between *S. grande* and *S. aromaticum* than *S. cumini* and *S. grande*, and *S. cumini* and *S. aromaticum* (**Supplementary Table 11**). Further, a higher percentage of *S. cumini* genes and a higher number of collinear blocks were present in the inter-species collinear blocks constructed between *S. cumini* and *S. grande*, compared to *S. cumini* and *S. aromaticum* (**Supplementary Table 11**). 17,882 *S. cumini* genes (29.22%) were present in the inter-species collinear blocks constructed with both *S. aromaticum* and *S. grande*, indicating their conserveness. The distribution of the 17,882 genes in KEGG pathways is mentioned in **Supplementary Table 12**.

**Figure 1:**
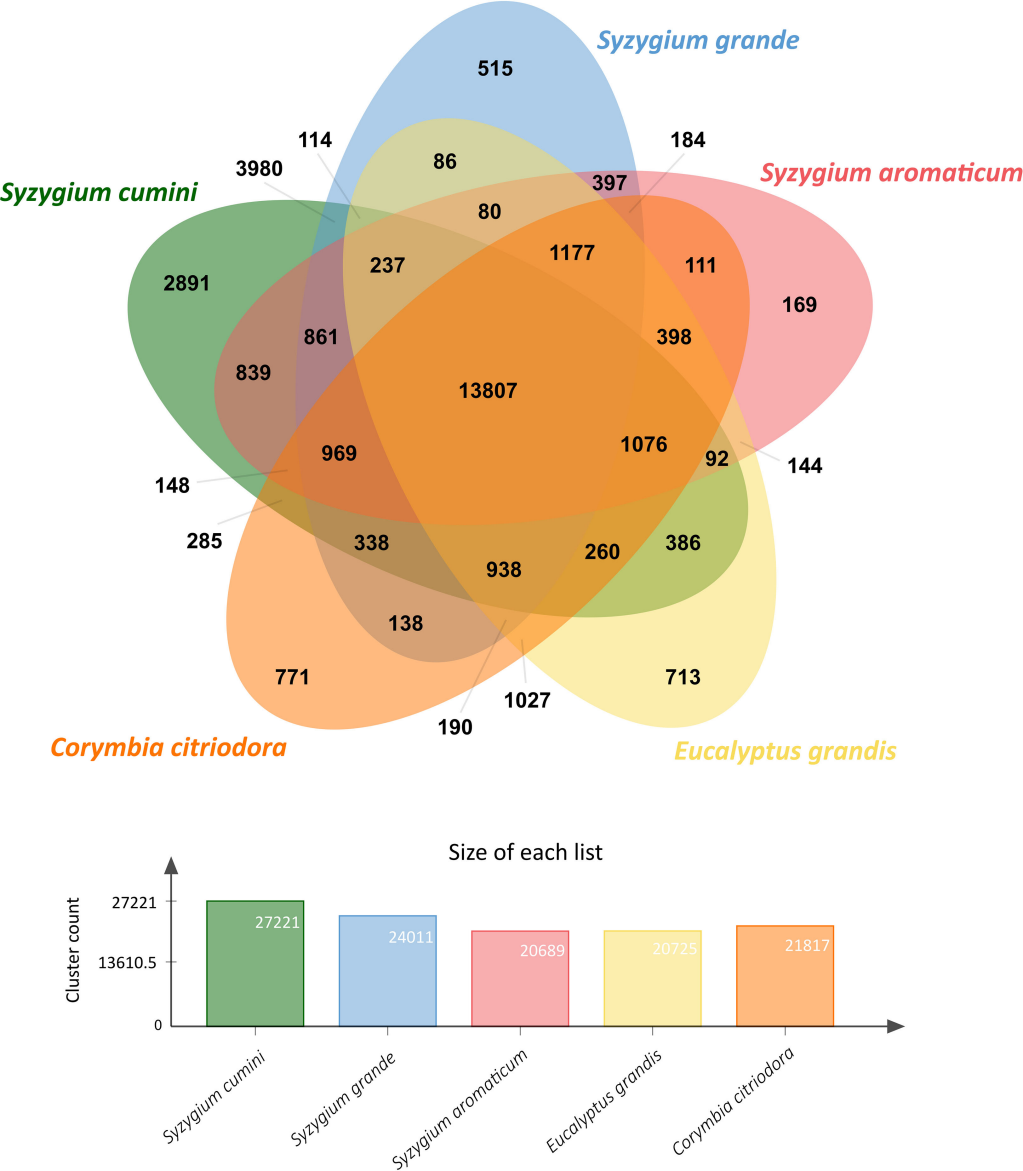
Orthologous gene clusters among *S. cumini* and other species from Myrtales plant order.

Gene clustering among *S. cumini* and four other species from Myrtales order showed a large number of species-specific gene clusters in *S. cumini* (2,891 clusters) compared to other species (**Figure 1**). 3,980 gene clusters were common between *S. cumini* and *S. grande*, and 839 common gene clusters were identified between *S. cumini* and *S. aromaticum*. Genes included in the species-specific gene clusters of *S. cumini* (15,721 genes) were involved in various KEGG pathways mentioned in **Supplementary Table 13**.

### Phylogenetic position of *S. cumini*

1,465 one-to-one fuzzy orthogroups were identified across 23 species spanning 18 Eudicot plant orders. Filtered and concatenated sequence alignments of the orthogroups containing 1,248,870 alignment positions were used to construct the species phylogenetic tree with *Zea mays* as the outgroup species.

**Figure 2:**
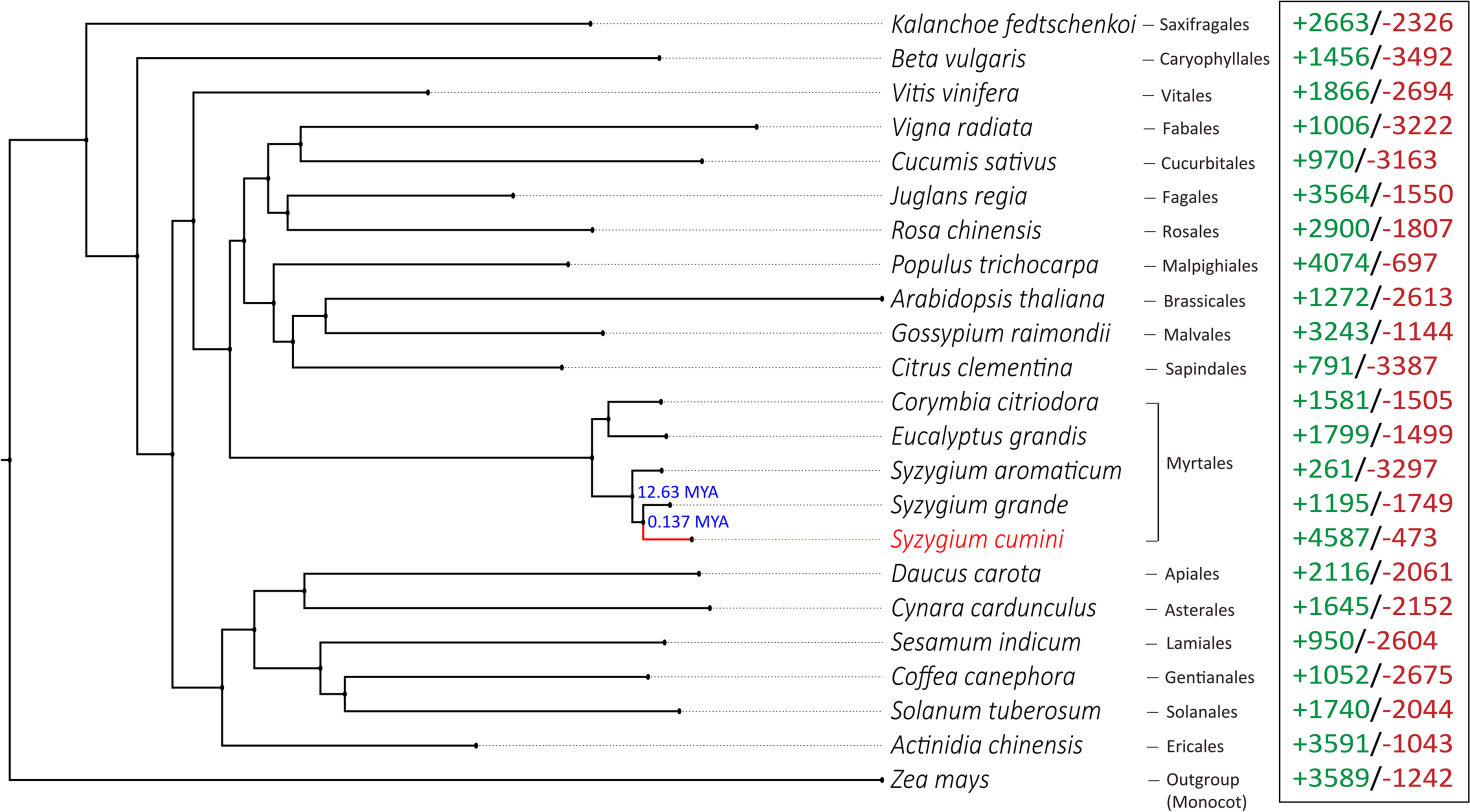
Phylogenetic position of *S. cumini* with respect to Eudicot species from Myrtales and 17 other plant orders. *Zea mays* was used as an outgroup species. Numbers mentioned in the nodes denote the divergence times of *Syzygium* species obtained from TimeTree v5 database (Kumar et al., 2022). Numbers in green and red represent the number of expanded and contracted gene families in each species, respectively.

In the phylogenetic tree, *S. cumini* was found in a position closer to *S. grande* (in the same clade) compared to *S. aromaticum* (**Figure 2**), which can further be explained by a higher number of collinear blocks and a higher number of shared gene clusters present between *S. cumini* and *S. grande*, compared to *S. cumini* and *S. aromaticum* (**Figure 1**; **Supplementary Table 11**). Among all the core Eudicot species in our phylogenetic tree, the species from the Saxifragales plant order (*K. fedtschenkoi*) diverged the earliest. The relative phylogenetic positions of the Eudicot orders were similar to the previous studies (Soltis et al., 2000; Chakraborty et al., 2022).

### Gene family evolution

A total of 17,366 filtered gene families were identified across 23 species. Among these, 4,587 gene families were expanded, and 473 gene families were contracted in *S. cumini* species. The number of expanded gene families was much higher than that of *S. grande* and *S. aromaticum* (**Figure 2**). Among the expanded gene families, 41 families were highly expanded (>25 expanded genes) in *S. cumini* (**Supplementary Table 14**). The highly expanded gene families were involved in secondary metabolism-related pathways, such as phenylpropanoid and flavonoid biosynthesis (**Supplementary Table 15**).

### Genes with evolutionary signatures

8,583 orthogroups across 13 species were constructed to identify the *S. cumini* genes with evolutionary signatures. 1,630 genes were positively selected (p-value < 0.05), 1,113 genes had unique amino acid substitution with functional impact, and 135 genes showed higher nucleotide divergence (**Supplementary Tables 16–18**). Among these genes, 430 genes had more than one signature of adaptive evolution (MSA genes). 333 of the MSA genes were also supported by gene expression (TPM > 1) data obtained in this study (**Supplementary Data 1**). GO categories of the *S. cumini* MSA genes are mentioned in **Supplementary Table 19**.

### Adaptive evolution of genes involved in secondary metabolism pathways

Plant secondary metabolites are derived from the primary metabolites and mainly function in the interaction of plants with their environment, abiotic and biotic stress tolerance, and are responsible for the medicinal properties of plants. The main classes of plant secondary metabolites are terpenoids, phenolic compounds, and alkaloids. The phenylpropanoid-flavonoid (PF) biosynthesis pathway is the key pathway for producing a wide range of phenolic compounds, such as flavonol, lignin, and anthocyanin (Taheri et al., 2022). Flavonoids and phenylpropanoids were the most abundant bioactive compounds in the fruit extracts of *S. cumini*, showing a wide range of pharmacological activities and can be used as preventive measures in many diseases, including type-2 diabetes (Correia et al., 2023).

#### PF biosynthesis pathway

**Figure 3:**
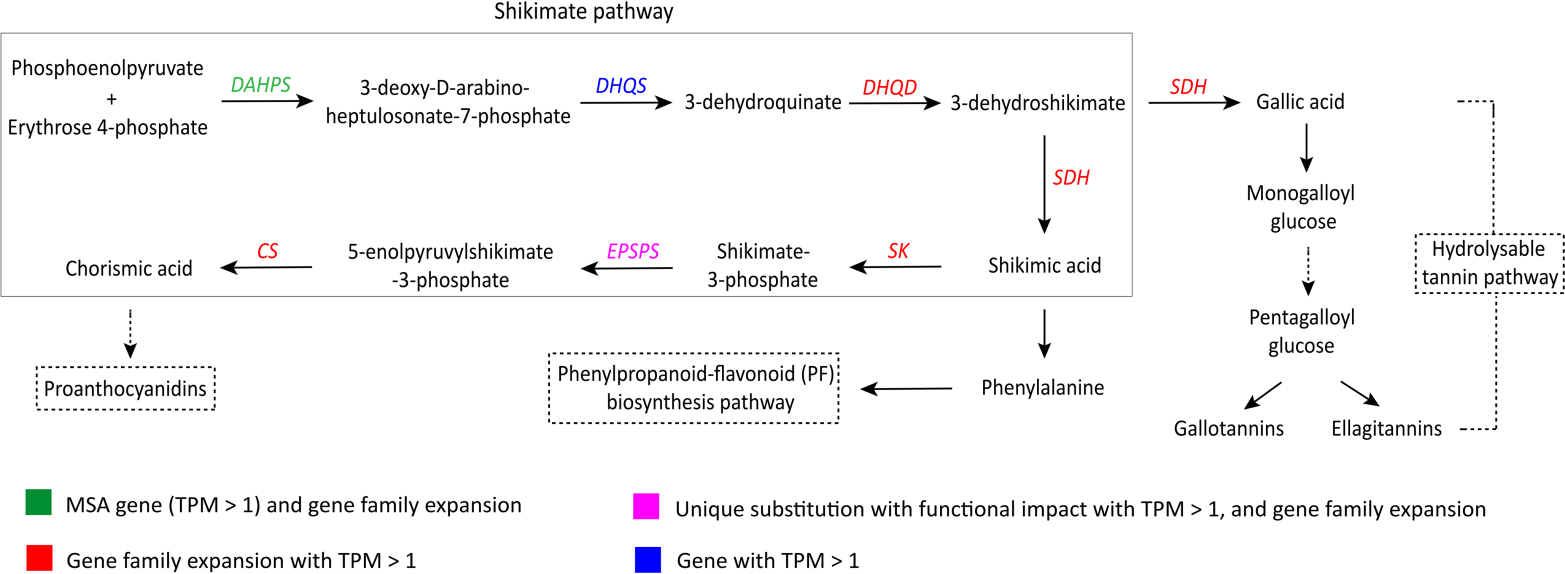
Adaptive evolution of the shikimate pathway in *S. cumini* (Salminen and Karonen, 2011; Mora et al., 2022). *DAHPS*, 3-deoxy-D-arabino-heptulosonate-7-phosphate synthase; *DHQS*, Dehydroquinate synthase; *DHQD*, 3-dehydroquinate dehydratase; *SDH*, Shikimate dehydrogenase; *SK*, Shikimate kinase; *EPSPS*, 5-enolpyruvylshikimate 3-phosphate synthase; *CS*, Chorismate synthase.

The shikimate pathway is responsible for the production of the precursors of phenylpropanoids along with tannins and other major groups of phenolic compounds (Salminen and Karonen, 2011). Genes involved in the shikimate pathway showed evolutionary signatures – *DAHPS* was identified as an MSA gene (TPM > 1), and *EPSPS* showed unique substitution with functional impact. Six (*DAHPS*, *DHQD*, *SDH*, *SK*, *EPSPS*, and *CS*) of the seven genes involved in the shikimate pathway also showed gene family expansion in *S. cumini* (**Figure 3**; **Supplementary Table 20**).

**Figure 4:**
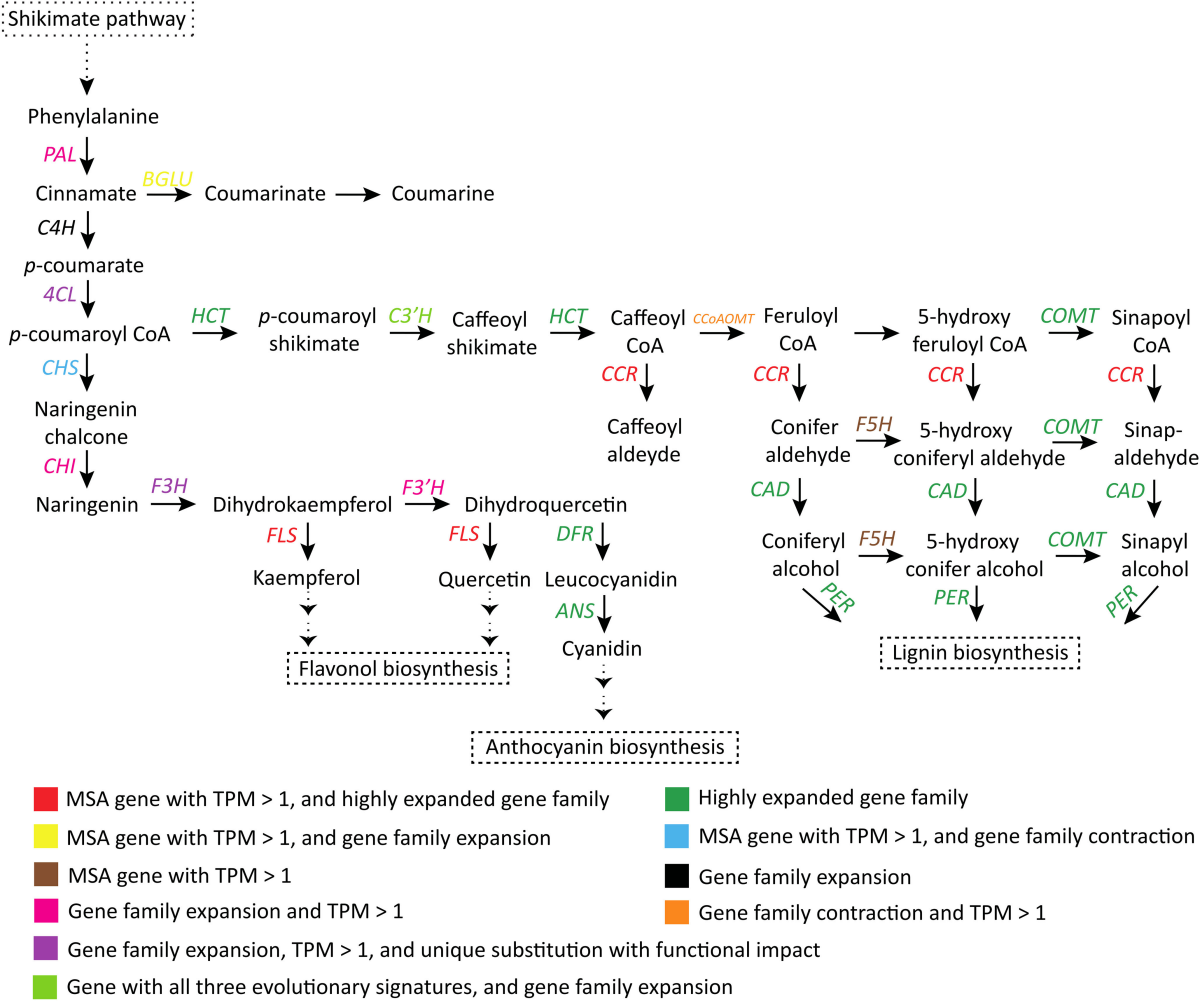
Adaptive evolution of the phenylpropanoid-flavonoid (PF) biosynthesis pathway (Yadav et al., 2020; Taheri et al., 2022). *PAL*, Phenylalanine ammonia lyase; *C4H*, Cinnamic acid 4-hydroxylase; *4CL*, 4-hydroxycinnamoyl-CoA ligase; *HCT*, Shikimate O-hydroxycinnamoyltransferase; *C3’H*, p-coumaroyl shikimate 3′-hydroxylase; *CCoAOMT*, Caffeoyl-CoA O-methyltransferase; *COMT*, Caffeic acid 3-O-methyltransferase; *CCR*, Cinnamoyl-CoA reductase; *F5H*, Ferulate-5-hydroxylase; *CAD*, Cinnamyl-alcohol dehydrogenase; *PER*, Peroxidase; *CHS*, Chalcone synthase; *CHI*, Chalcone isomerase; *F3H*, Flavanone 3-hydroxylase; *F3’H*, Flavonoid 3′-monooxygenase; *FLS*, Flavonol synthase; *DFR*, Dihydroflavonol 4-reductase; *ANS*, Anthocyanidin synthase.

In the common phenylpropanoid pathway (conversion of phenylalanine to *p*-coumaroyl CoA), all three genes *PAL*, *C4H* and *4CL* showed gene family expansion. *PAL* and *4CL* genes were highly expressed (TPM > 1), and *4CL* also showed unique substitution with functional impact. In the downstream phenylpropanoid pathway, *HCT*, *COMT*, *CAD*, *CCR*, and peroxidase genes had highly expanded gene families (with >25 expanded genes). *CCR* and *F5H* were identified as MSA genes (TPM > 1). Peroxidase and *CAD* genes showed unique substitution with functional impact. *C3’H* showed all three evolutionary signatures and gene family expansion, and *CCoAOMT* gene family was contracted (**Figure 4**; **Supplementary Table 20**).

*p*-coumaroyl CoA, formed in the phenylpropanoid pathway, is also a precursor to flavonoid (e.g., flavonol and anthocyanin) and lignin biosynthesis. Enzymes involved in the biosynthesis steps were also adaptively evolved in *S. cumini* species. *FLS* and *CHS* were found among the MSA genes (TPM > 1), and gene families of these two genes showed high expansion and contraction, respectively. Gene families of *CHI*, *F3’H*, and *F3H* were expanded, and *F3H* also had unique substitution with functional impact. All these genes had high gene expression (TPM > 1). Further, gene families of *DFR* and *ANS*, the two major enzymes for anthocyanin biosynthesis, were highly expanded in *S. cumini* (**Figure 4**).

#### Terpenoid biosynthesis pathway

**Figure 5:**
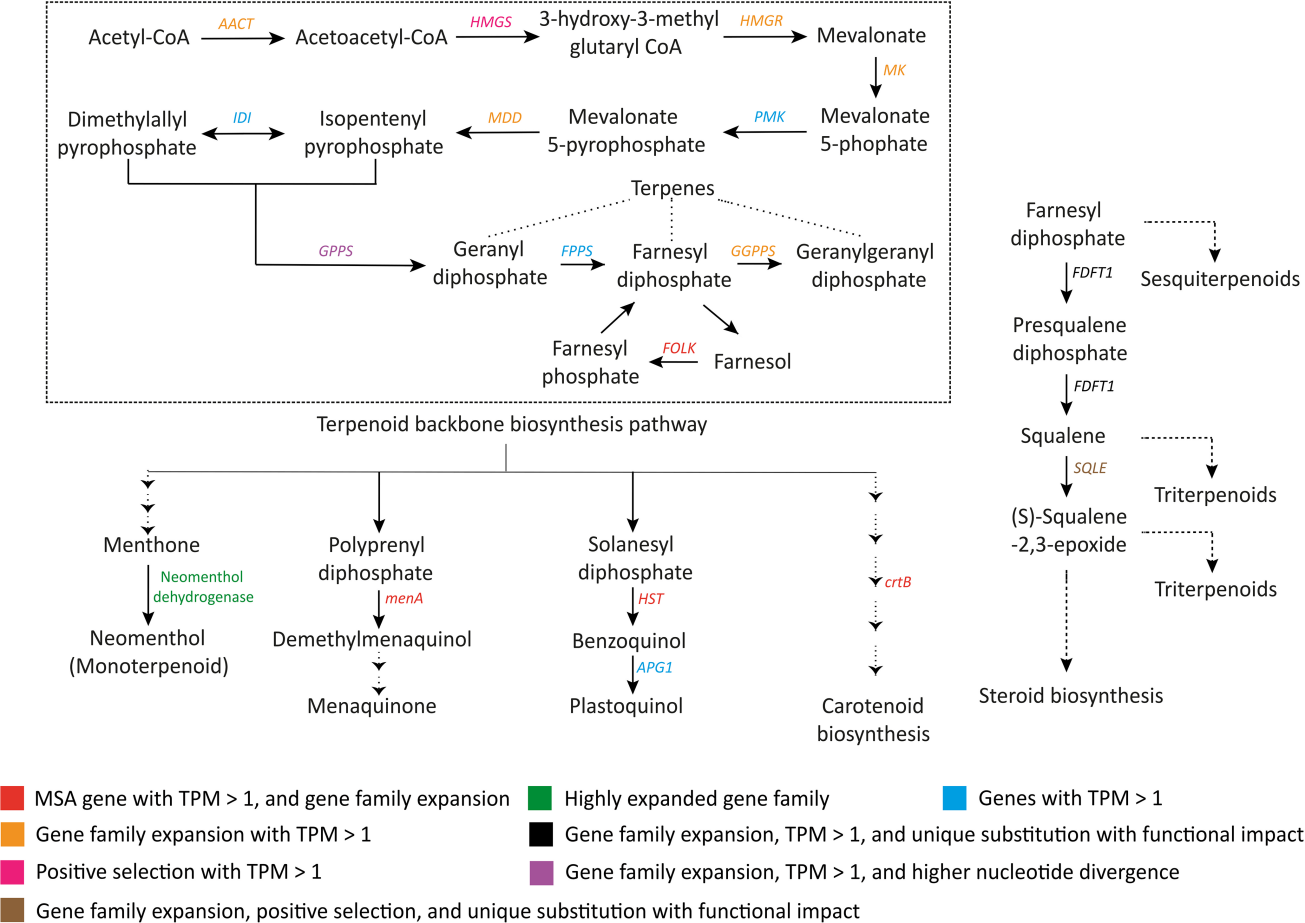
Evolutionary signatures in terpenoid and other terpenoid-quinone biosynthesis genes. *AACT*, Acetoacetyl-CoA thiolase; *HMGS*, HMG-CoA synthase; *HMGR*, HMG-CoA reductase; *MK*, Mevalonate kinase; *PMK*, Phosphomevalonate kinase; *MDD*, Mevalonate-5-diphosphate decarboxylase; *IDI*, Isopentenyl diphosphate isomerase; *GPPS*, Geranyl diphosphate synthase; *FPPS*, Farnesyl diphosphate synthase; *GGPPS*, Geranylgeranyl diphosphate synthase; *FOLK*, Farnesol kinase; *menA*, 1;4-dihydroxy-2-naphthoate polyprenyltransferase; *HST*, Homogentisate solanesyltransferase; *APG1*, MPBQ/MSBQ methyltransferase; *crtB*, 15-cis-phytoene synthase; *FDFT1*, Farnesyl-diphosphate farnesyltransferase 1; *SQLE*, Squalene monooxygenase.

Terpenoids are another important class of plant secondary metabolites. Fruits and flowers of *S. cumini* are rich in terpenoids (Chaudhary and Mukhopadhyay, 2012; Correia et al., 2023), and the terpenoids present in the *S. cumini* leaves can be used to treat inflammatory diseases (Siani et al., 2013). In support of this, adaptive evolution in the genes involved in terpenoid backbone biosynthesis pathways was observed. *AACT*, *HMGR*, *MK*, *MDD*, *GPPS*, and *GGPPS* had expanded gene families (**Supplementary Table 20**). Further, *HMGS* was positively selected, and *GPPS* showed higher nucleotide divergence in *S. cumini*. *FOLK* gene, responsible for forming farnesyl diphosphate from farnesol, was found among the MSA genes (TPM > 1) along with gene family expansion. Further, the terpenoid backbone biosynthesis pathway is the precursor to the formation of monoterpenes, triterpenes, sesquiterpenes, other terpenoid-quinones, carotenoids, etc. Enzymes involved in these pathways such as *FDFT1*, *SQLE*, neomenthol dehydrogenase, *menA*, *HST*, and *crtB* also showed adaptive evolution in *S. cumini* species (**Figure 5**).

**Figure 6:**
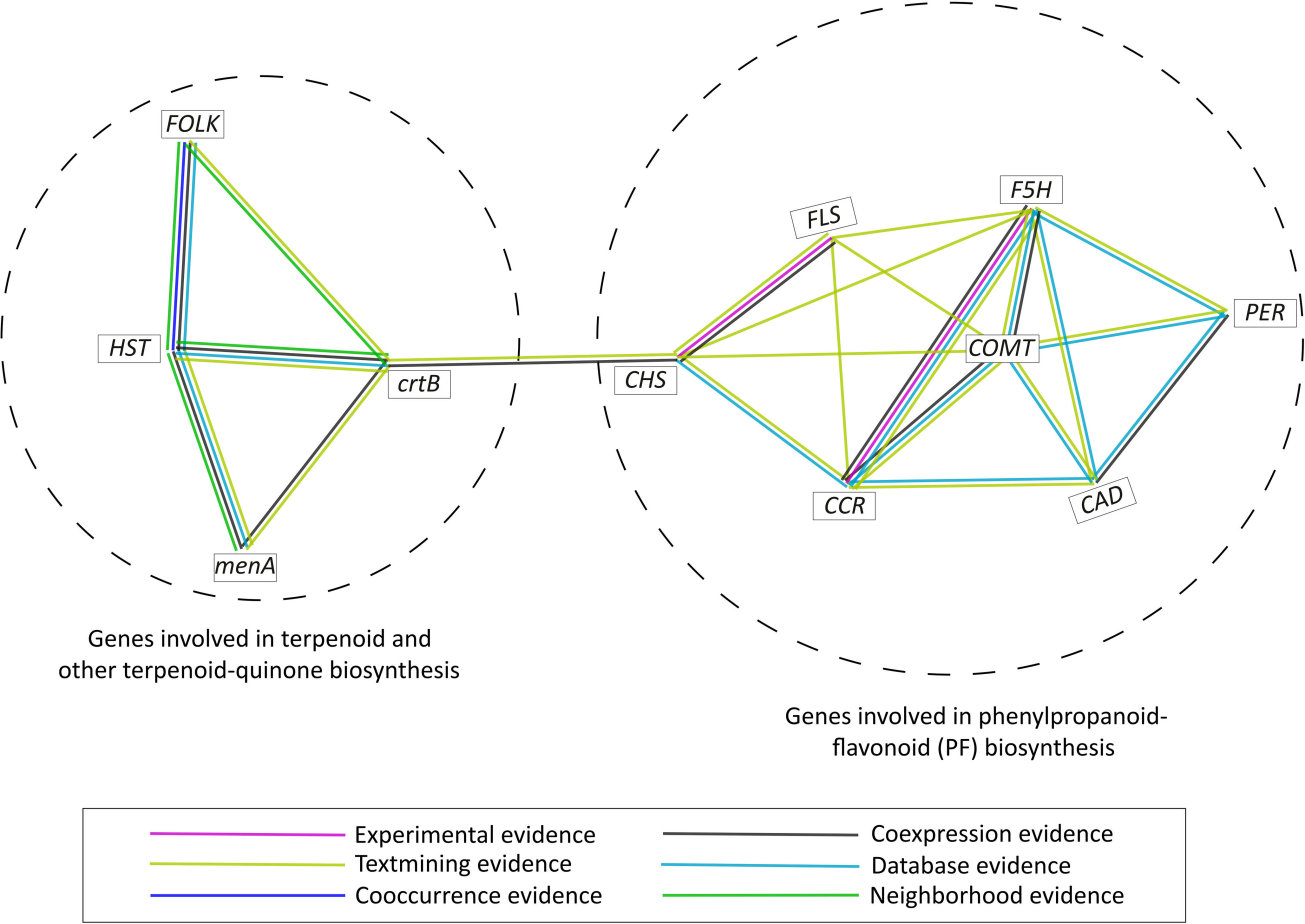
Protein-protein interaction among the MSA genes (TPM > 1) and highly expanded gene families involved in phenylpropanoid-flavonoid (PF) biosynthesis and terpenoid biosynthesis pathways in *S. cumini*.

Using STRING database (v11.5) (Szklarczyk et al., 2021), protein-protein interaction was examined in the genes belonging to phenylpropanoid-flavonoid (PF) pathway and terpenoid biosynthesis pathway. Only the MSA genes (with TPM > 1) and the genes in highly expanded genes families of *S. cumini* were considered for the above analysis. Based on the protein-protein interaction evidence available on the STRING database, two clusters were formed by the terpenoid and phenolic compounds biosynthesis-related genes, and an association between these clusters was observed mediated by *CHS* (involved in PF biosynthesis pathway) and *crtB* (involved in terpenoid biosynthesis) indicating a functional relationship between the two pathways (**Figure 6**).

#### Alkaloid and other secondary metabolites biosynthesis

**Figure 7:**
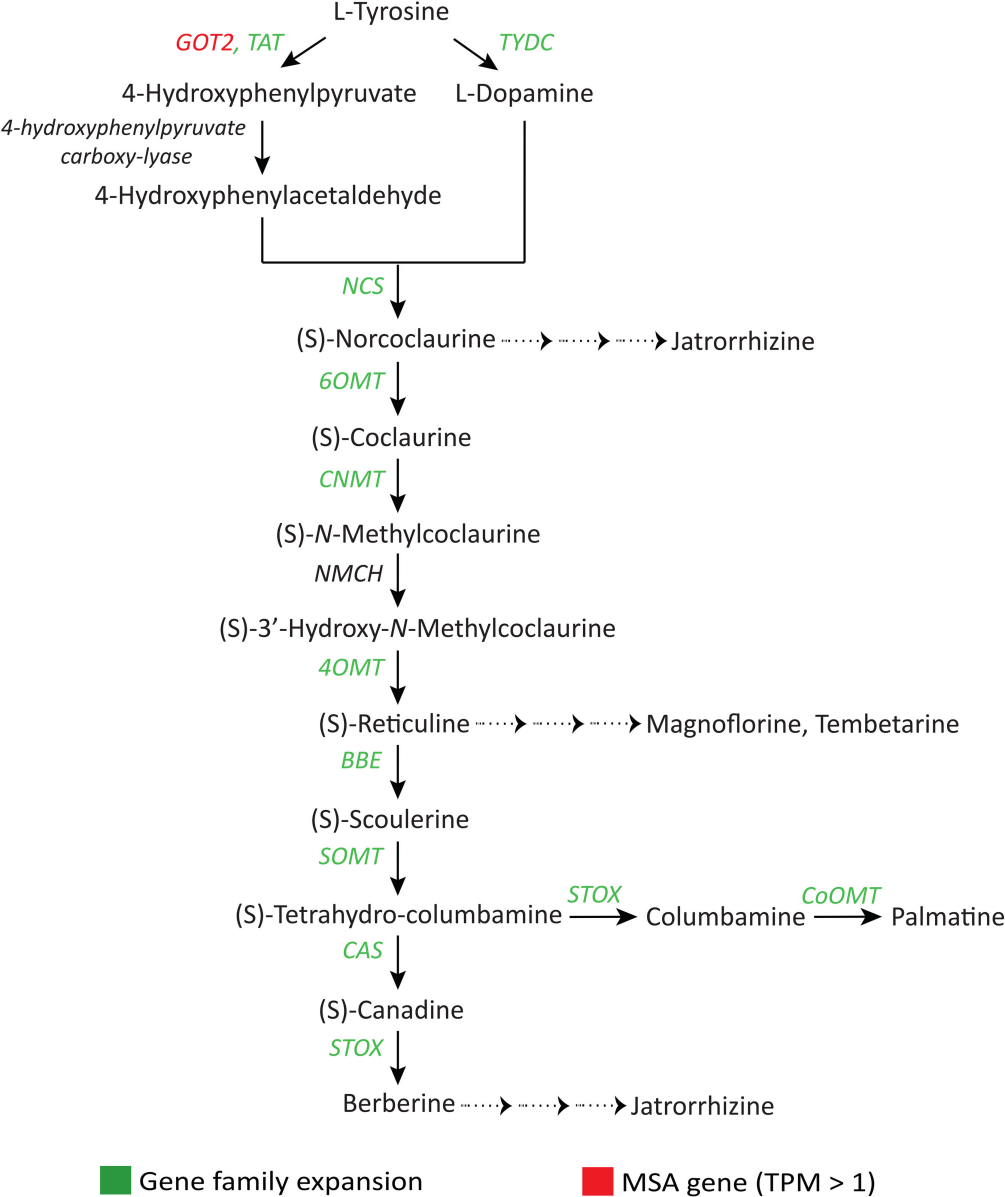
Adaptive evolution of genes involved in the benzylisoquinoline alkaloid (BIA) biosynthesis pathway (Deng et al., 2018). *GOT2*, Aspartate aminotransferase; *TAT*, Tyrosine aminotransferase; *TYDC*, Tyrosine decarboxylase; *NCS*, (S)-Norcoclaurine synthase; *6OMT*, Norcoclaurine 6-O-methyltransferase; *CNMT*, (S)-Coclaurine N-methyltransferase; *NMCH*, (S)-N-Methylcoclaurine 3’-hydroxylase; *4OMT*, 3’-Hydroxy-N-methylcoclaurine 4’-O-methyltransferase; *BBE*, Berberine bridge enzyme; *SOMT*, (S)-Scoulerine 9-O-methyltransferase; *CAS*, (S)-Canadine synthase; *STOX*, (S)-Tetrahydroprotoberberine oxidase; *CoOMT*, Columbamine O-methyltransferase.

The pharmacological activities of the alkaloids provide essential health benefits through the fruits and other plant parts of *S. cumini* (Srivastava and Chandra, 2013). Genes (*TAT*, *TYDC*, *NCS*, *6OMT*, *CNMT, 4OMT, BBE, SOMT, CAS, STOX,* and *CoOMT*) involved in the benzylisoquinoline alkaloid (BIA) biosynthesis pathway, a diverse group of alkaloids with numerous medicinal properties, showed gene family expansion (**Figure 7**, **Supplementary Table 20**). *GOT2* gene involved in isoquinoline and tropane alkaloid biosynthesis pathways was found among the MSA genes (with TPM > 1) (Zhou and Chen, 2022). Further, *AAE* gene family and another ‘GDSL’ lipolytic family involved in indole alkaloid biosynthesis were highly expanded (Ruppert et al., 2005).

Among other notable adaptively evolved genes involved in secondary metabolites biosynthesis, *F6H* (highly expanded gene family) functions in scopoletin biosynthesis (Kai et al., 2008), *PRR1* (MSA gene with TPM > 1) helps in lignan biosynthesis (Hemmati et al., 2007), and *BX1* (MSA gene with TPM > 1) is involved in glucoside (via benzoxazinoid biosynthesis pathway) production (Frey et al., 2009).

### Plant BGCs in *S. cumini* genome

**Figure 8:**
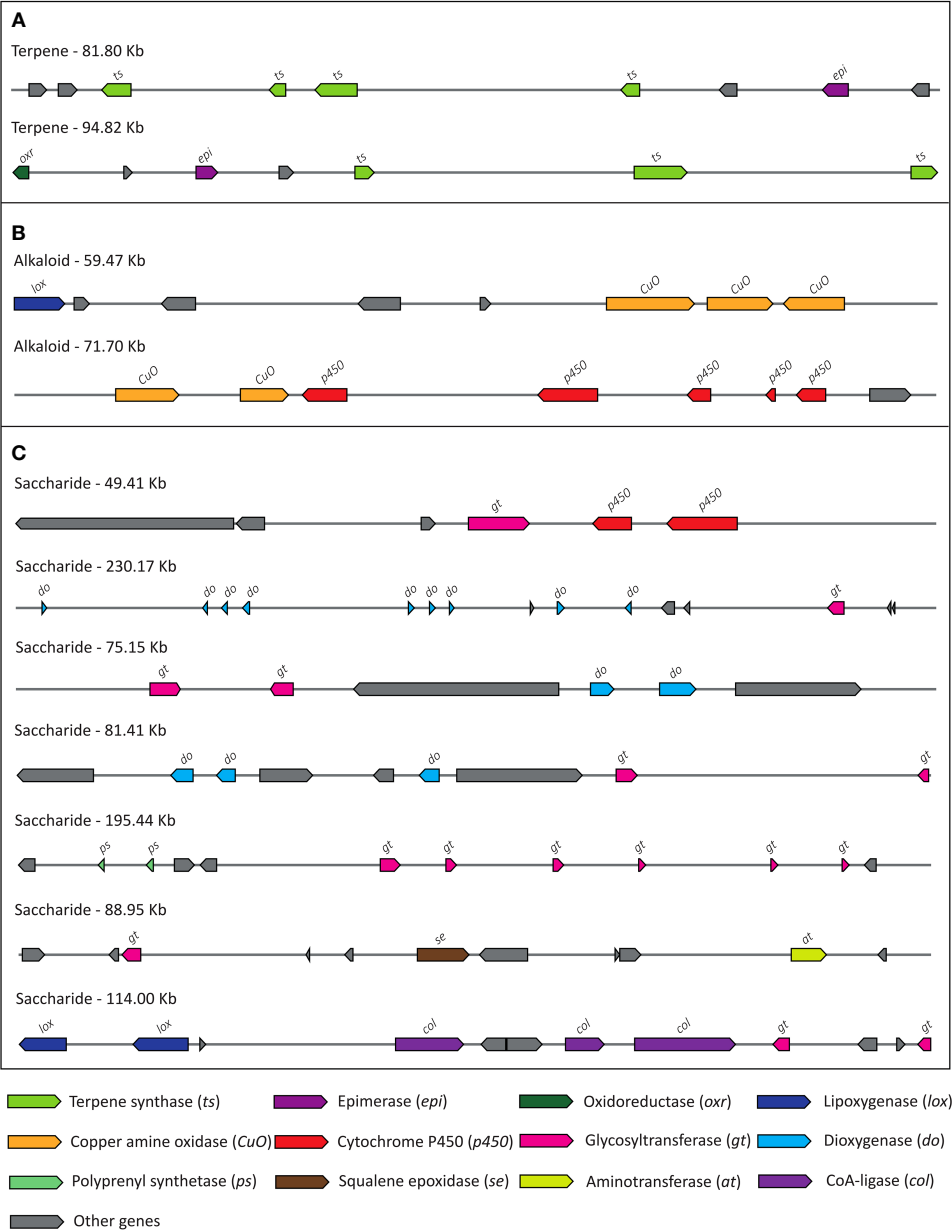
The biosynthetic gene clusters (BGCs) annotated in *S. cumini* genome. **(A)** Terpene gene clusters, **(B)** Alkaloid gene clusters, **(C)** Saccharide gene clusters.

Plants produce immensely diverse specialized metabolites, such as secondary metabolites, that function in ecological interactions and possess nutritional and medicinal importance. Genes encoding these biosynthetic pathways are often clustered in a genomic locus known as the biosynthetic gene cluster (BGC) (Kautsar et al., 2017). 39 BGCs were identified in *S. cumini* genome containing a total of 562 plant biosynthetic genes, which were involved in KEGG pathways such as phenylpropanoid biosynthesis, cell cycle, plant hormone signal transduction, terpenoid biosynthesis, etc. (**Figure 8**; **Supplementary Table 21**). Among the *S. cumini* genes in the BGCs, 29 key genes involved in secondary metabolites biosynthesis were present in one or more copies, and 35 genes showed at least one of the three signatures of adaptive evolution, namely, positive selection, higher nucleotide divergence, and unique substitution with functional impact (**Supplementary Tables 22, 23**). However, not all secondary metabolite biosynthesis pathway genes are present in clusters; therefore, the biosynthetic genes that are not present in clusters will not be identified in the BGCs (Shi and Xie, 2014).

### Adaptive evolution of genes associated with stress tolerance mechanisms

MSA genes of *S. cumini* were also involved in various biotic (such as pathogen resistance and defense against herbivores) and abiotic (such as ROS scavenging, heat, drought, and salinity, etc.) stress tolerance mechanisms (**Supplementary Data 2**). Among the key genes with MSA involved in biotic stress tolerance responses, *GI* downregulates salicylic acid accumulation and alters the phenylpropanoid pathway, thus reducing PR (Pathogenesis-Related) gene expression and negatively affecting biotic defense responses (Kundu and Sahu, 2021). *BSK* provides resistance against bacterial and fungal pathogens by playing a role in pattern-triggered immunity (PTI) (Majhi et al., 2019), *NPR1* is a crucial regulator of salicylic acid signaling and triggers immune responses by inducing PR genes (Chen et al., 2019), *MPK3* responds to biotic stress by upregulating jasmonic acid signaling and negatively regulating salicylic acid accumulation (Jagodzik et al., 2018), *PIK1* also acts in pathogen recognition and activation of defense responses (Romeis, 2001).

Among the major genes with MSA involved in abiotic stress tolerance responses, *ABF* regulates the expression of abscisic acid-responsive genes to provide salinity, drought, and osmotic stress tolerance to plants (Feng et al., 2019), *MPAO* facilitates oxidative burst-mediated programmed cell death to aid plant defense responses (Yoda et al., 2006), *KUP* K^+^ transporter family is involved in potassium deficiency and salt and drought stress response (Yang et al., 2020), Heat shock transcription factor (Hsf) regulates oxidative stress response by directly sensing the reactive oxygen species (ROS) (Miller and Mittler, 2006). Besides these, *LOX* confers abiotic (drought, salinity, etc.) and biotic stress tolerance (Viswanath et al., 2020), and *CNGC* has multifaceted functions in plants, such as pathogen resistance and abiotic (salt, drought, cold, etc.) stress tolerance (Guo et al., 2018).

## Discussion

In this study, we performed whole genome sequencing of *S. cumini* species and constructed a draft genome assembly for the first time. It is only the third and till date the largest genome to be sequenced from the largest tree genus containing approximately 1,200 species. *S. cumini* was previously reported to show intraspecific polyploidy compared to *S. aromaticum* and *S. grande* (Ohri, 2015). Our analyses using two independent approaches to estimate the genomic ploidy also confirmed the tetraploidy in *S. cumini* genome. Further, the genome was found to be highly heterozygous (3.25%), and a combination of polyploidy and high heterozygosity increases the genomic complexity in this species. Polyploidy causes difficulty in haplotype resolving (Kyriakidou et al., 2018) and a higher percentage of allelic differences (1% or above) also poses a challenge in genome assembly (Asalone et al., 2020). Despite of this genomic complexity, we could successfully construct the whole genome assembly of *S. cumini* with the assembled genome size close to the predicted genome size.

We used multiple approaches such as BUSCO assessment, LAI score estimation, and read mapping percentage calculation to evaluate the genome assembly quality. 98.3% complete BUSCOs in the genome assembly suggest a near-complete genome assembly. LAI score of 11.69 indicates that the genome assembly constructed in this study can be considered as a “Reference” quality assembly (Ou et al., 2018). LAI score of *S. cumini* genome constructed in this study was also similar to the other chromosome-scale plant genome assemblies such as *Angelica sinensis* (Han et al., 2022). A high percentage of mapped reads onto the genome assembly further attests to the assembly quality. Further, the usage of strict parameters, AED cut-off <0.5, and coding gene length ≥150 bp in the MAKER pipeline underscores the quality of the high-confidence coding genes. The presence of 92.8% BUSCOs in the coding gene set also suggests the near-completeness of the genome annotation performed in this study. Further, the complete structures (exon-intron number and gene length) of the *S. cumini* key genes involved in PF biosynthesis and terpenoid biosynthesis pathways could be identified, which was similar to the other two high-quality genome assemblies of *Syzygium* species, that also attests to the quality of *S. cumini* genome assembly (**Supplementary Tables 24, 25**, **Supplementary Text**).

We also noted a high percentage of complete and duplicated BUSCOs (D score) in the genome assembly and coding gene set of *S. cumini* (**Supplementary Table 3**), which is perhaps due to the additional neopolyploidy event following the Pan-Myrtales WGD event in *S. cumini* species (tetraploid) compared to the other *Syzygium* species that remained at the same ploidy level (Low et al., 2022). This event could also be the reason for an increased genome size of *S. cumini* compared to *S. grande* and *S. aromaticum* (Low et al., 2022; Ouadi et al., 2022). The increase in genome size also appears to be due to an expansion in copy number (37% higher) of LTR-RT repeat elements in *S. cumini* genome compared to *S. grande*, and an overall 6.4% and 8.1% higher repeat content compared to *S. grande* and *S. aromaticum* genomes, respectively (**Supplementary Table 5**) (Low et al., 2022; Ouadi et al., 2022; Zhu et al., 2023). The neopolyploidy event in *S. cumini* might also be the cause of a greater number of coding genes (61,195 genes), a greater fraction of genes (90.55%) originated from duplicated events, a higher number of gene clusters (27,221), and a higher number of species-specific gene clusters (2,891) observed in *S. cumini* species compared to *S. grande* and *S. aromaticum* (**Figure 1**). Duplicated genes may either undergo deletion or pseudogenization due to relaxed selection pressure (Wang et al., 2018) or acquire novel functions (Panchy et al., 2016), which could also be the case in *S. cumini* species as observed in the adaptive evolution of secondary metabolism pathways.

Further, the consideration of species from Myrtales order (including two other *Syzygium* species) and species from its closer phylogenetic orders for comparative analysis to identify the genes with evolutionary signatures in *S. cumini* helped to reduce the false positives that could have resulted from the greater genetic distance of the selected species. The genes with evolutionary signatures identified from genomic analyses were also supported by transcriptomic analysis. It is important to note that MSA genes and the genes from highly expanded gene families were found to be majorly involved in secondary metabolite biosynthesis pathways such as phenylpropanoids, flavonoids, alkaloids, and terpenoids, which are responsible for the medicinal properties of this species.

Phenylpropanoids play essential roles in plant development, response to abiotic and biotic stress signals, and biosynthesis of a broad spectrum of secondary metabolites (Vogt, 2010). Phenylpropanoid-derived metabolites contribute to the biosynthesis of several other secondary metabolites, such as lignin and lignan, isoflavonoid, coumarin, stilbene, anthocyanin, isoquercetin, myrecetin, and kaemferol, which confer numerous pharmacological properties in *S. cumini* species (Ayyanar and Subash-Babu, 2012; Chaudhary and Mukhopadhyay, 2012; Srivastava and Chandra, 2013). One particular class of flavonoids - anthocyanin, is responsible for the purple-black color of the fruits of *S. cumini* and their health benefits (Chaudhary and Mukhopadhyay, 2012). Phenolic compounds (e.g., catechin, gallic acid, etc.) extracted from *S. cumini* seeds have immense potential as anti-diabetic and anti-oxidant agents, that have found commercial significance as nutraceutical ingredients in modern medicine and can be used as a substitution of allopathic remedies for chronic diseases such as type-2 diabetes (Mahindrakar and Rathod, 2021; Kumar et al., 2023). One of the main findings of this study was the identification of evolutionary signatures and gene family evolution of all the key *S. cumini* genes involved in the PF biosynthesis pathway (**Figure 4**). It is an important finding because the evolutionary signatures and evolution in gene families have been recognized as critical mechanisms shaping natural variation for species adaptation, which might also be the case in this species (Guo, 2013; Chakraborty et al., 2021). Further, 741 genes were present in the expanded gene families of the PF biosynthesis pathway, among which 98.9% of the genes originated from different modes of duplication, which function in increasing the dosage of gene products and in accelerating the metabolic flux for rate-limiting steps in such biosynthetic pathways (Conant and Wolfe, 2007; Bekaert et al., 2011). Taken together, the adaptive evolution of PF biosynthesis pathway and its precursor shikimate pathway in *S. cumini* could be responsible for their numerous therapeutic properties, specifically the anti-diabetic property conferred by the seeds and leaves (Srivastava and Chandra, 2013).

Notably, the comparative evolutionary analyses revealed 14 key genes involved in the biosynthesis of terpenoids and other terpenoid-quinone compounds to show adaptive evolution in *S. cumini* (**Figure 5**; **Supplementary Data 1**). Terpenoids are a structurally diverse class of secondary metabolites responsible for plant defense responses against herbivores and pathogens, and are abundant in *S. cumini* fruits contributing to the anti-oxidant and anti-inflammatory properties (Cheng et al., 2007). Other terpenoid-quinone compounds also function in plant stress tolerance responses (Liu and Lu, 2016) and show pharmacological activities (Gordaliza, 2012). Thus, the adaptive evolution of terpenoid biosynthesis pathway could explain the anti-inflammatory properties of *S. cumini* leaves and seeds conferred by the terpenoids (Siani et al., 2013; Srivastava and Chandra, 2013).

Among the other classes of secondary metabolites, alkaloids present in different plant parts of *S. cumini* are pharmaceutically diverse secondary metabolites with curative properties against many diseases (Ziegler and Facchini, 2008). Alkaloids, along with flavonoids and tannins also confer anti-arthritic property to the *S. cumini* seeds (Srivastava and Chandra, 2013). Glucosides are also critical secondary metabolites for plant defense responses and possess therapeutic properties (Bennett and Wallsgrove, 1994). *S. cumini* seed extracts contain alkaloid jambosine, and glucoside jambolin that prevents the conversion step of starch into sugar (anti-diabetic), which is the most significant therapeutic property of this species (Ayyanar and Subash-Babu, 2012). In this study, genes related to alkaloid and glucoside biosynthesis (e.g., *GOT2*, *AAE*, *BX1*, etc.) showed adaptive evolution in *S. cumini* that emphasizes the genomic basis for its pharmacological properties.

It is important to mention that the secondary metabolites are produced and regulated in response to various abiotic and biotic stresses, and aid in better survival of the plants and confer their medicinal properties (Isah, 2019). Here, we also noted that various biotic and abiotic stress tolerance response genes displayed multiple signatures of adaptive evolution in *S. cumini* (**Supplementary Data 2**). Further, KEGG pathways related to phenylpropanoid, flavonoid, terpenoid, alkaloid biosynthesis, plant hormone signal transduction, and plant-pathogen interaction were found in all the gene sets that showed the evolutionary signatures and gene family expansion (**Supplementary Tables 15–18**; **Supplementary Data 1**). Taken together, it is tempting to speculate that the adaptive evolution of major plant secondary metabolism pathways in *S. cumini* species confers unprecedented anti-diabetic, anti-oxidant, anti-inflammatory, and other pharmacological properties of this tree. Further, the whole genome sequence of *S. cumini* will facilitate future genomic, evolutionary, and ecological studies on the world’s largest tree genus.

## Data availability statement

The original contributions presented in the study are included in the article/**Supplementary Material**, further inquiries can be directed to the corresponding author. Raw reads obtained from the genome and transcriptome sequencing have been deposited in NCBI SRA database under the BioProject accession - PRJNA982613 and BioSample accession - SAMN35711647.

## Author contributions

AC: Conceptualization, Data curation, Formal Analysis, Investigation, Methodology, Validation, Visualization, Writing – original draft, Writing – review & editing. SM: Methodology, Validation, Writing – original draft, Writing – review & editing. MSB: Data curation, Investigation, Writing – review & editing. VKS: Conceptualization, Funding acquisition, Investigation, Methodology, Project administration, Resources, Supervision, Validation, Writing – original draft, Writing – review & editing.
