# Supplementary material for: Genome sequencing of Syzygium cumini (jamun) reveals adaptive evolution in secondary metabolism pathways associated with its medicinal properties
Source: Front Plant Sci. 2023 Nov 17;14:1260414. doi: 10.3389/fpls.2023.1260414 (PMC10693344; doi:10.3389/fpls.2023.1260414)
Supplement: Supplementary file 1 [file DataSheet_1.pdf]

Title: Genome sequencing of *Syzygium cumini* (Jamun) reveals adaptive evolution in secondary metabolism pathways associated with its medicinal properties

Authors: Abhisek Chakraborty, Shruti Mahajan, Manohar S. Bisht, Vineet K. Sharma*

Affiliation:

MetaBioSys Group, Department of Biological Sciences, Indian Institute of Science Education and Research Bhopal, Bhopal, India

*Corresponding Author email:

Vineet K. Sharma - vineetks@iiserb.ac.in

E-mail addresses of authors:

Abhisek Chakraborty - abhisek18@iiserb.ac.in, Shruti Mahajan - shruti17@iiserb.ac.in, Manohar S. Bisht - manohar21@iiserb.ac.in, Vineet K. Sharma - vineetks@iiserb.ac.in

SUPPLEMENTARY TABLES

Supplementary Table 1. Summary of the raw genomic and transcriptomic data generated in this study for *S. cumini* species

Sequencing data	Total data (bases)	No. of reads	Average read length	Sequencing coverage*
Oxford Nanopore	14,406,638,579	3,035,671	4,746 bases	19.7X
10x Genomics	120,702,164,172	759,133,108	159 bases	165.3X
RNA-Seq data	15,098,162,682	94,956,998	159 bases	-

*Sequencing coverage was calculated based on the estimated genome size of 730.3 Mbp

Supplementary Table 2. Genome assembly statistics of *S. cumini* after each step of the assembly process

Parameters	Genome assembly stages						
	Canu	Pilon-polished	AGOUTI-scaffolded	ARCS-scaffolded	LINKS-scaffolded	Gap-closed	Final (≥5 Kbp)
No. of contigs	9,704	9,704	9,590	7,817	7,733	7,733	7,702
No. of contigs (≥ 10 Kb)	9,517	9,519	9,408	7,636	7,559	7,559	7,558
No. of contigs (≥ 25 Kb)	7,896	7,920	7,839	6,203	6,159	6,159	6,160
No. of contigs (≥ 50 Kb)	4,501	4,531	4,516	3,649	3,644	3,644	3,642
Total length	706,915,755	709,728,177	709,842,177	710,019,477	710,121,380	710,123,498	709,916,230
Total length (≥ 10 Kb)	705,558,535	708,387,522	708,517,385	708,702,544	708,862,130	708,864,248	708,761,311
Total length (≥ 25 Kb)	675,646,986	678,921,987	679,595,341	682,440,553	683,167,848	683,169,966	683,107,220
Total length (≥ 50 Kb)	550,185,570	553,802,139	556,960,263	589,112,729	591,191,411	591,193,547	591,013,944
Largest contig (bases)	1,351,405	1,345,917	1,345,917	1,571,180	1,571,180	1,571,180	1,571,886
N50 (bases)	102,070	102,402	104,735	176,662	179,158	179,158	179,217
L50	1,666	1,668	1,635	984	973	973	973
GC%	40.50	40.44	40.44	40.44	40.44	40.44	40.44
N's per 100 kbp	0.00	0.00	16.06	41.03	55.37	22.54	21.94
BUSCO completeness	95.4%	98.1%	98.2%	98.3%	98.1%	98.1%	98.3%

Supplementary Table 3. BUSCO statistics of genome assembly and coding gene set

Parameters	Genome assembly	Coding gene set
Complete and single-copy (S)	1,042 (64.6%)	833 (51.6%)
Complete and duplicated (D)	544 (33.7%)	437 (27.1%)
Fragmented (F)	9 (0.6%)	228 (14.1%)
Missing (M)	19 (1.1%)	116 (7.2%)
Total BUSCO groups searched	1,614	1,614

Supplementary Table 4. Chloroplast genome assembly statistics of *S. cumini* and comparison with the previous studies

Parameters	*S. cumini* (This study)	*S. cumini* (Asif et al., 2013)	*S. malaccense* (Tao et al., 2020)
Genome size	158,509 bases	160,373 bases	158,954 bases
Size of IR (Inverted Repeat) region	26,077 bases	26,392 bases	26,085 bases
Size of LSC (Large Single Copy) region	88,007 bases	89,081 bases	87,991 bases
Size of SSC (Small Single Copy) region	18,348 bases	18,508 bases	18,793 bases
No. of protein-coding genes	83	83	84
tRNA genes	37	37	37
rRNA genes	8	8	8
GC-content	37%	36.83%	36.97%

Supplementary Table 5. Summary statistics of repetitive regions of *S. cumini* genome identified using RepeatMasker

Total length (bases)		709,916,230			
GC%		40.44%			
Bases masked		365,690,999 (51.51%)			
			No. of elements	Length occupied (bp)	% of sequence
Retroelements			108,142	111,738,717	15.74
	SINEs		295	94,441	0.01
	LINEs		15,596	11,684,105	1.65
		L2/CR1/Rex	1,009	330,328	0.05
		R2/R4/NeSL	117	61,009	0.01
		RTE/Bov-B	799	263,126	0.04
		L1/CIN4	13,327	10,922,390	1.54
	LTR elements		92,251	99,960,171	14.08
		BEL/Pao	259	84,679	0.01
		Ty1/Copia	42,835	38,095,712	5.37
		Gypsy/DIRS1	44,452	57,410,705	8.09
		Retroviral	73	33,096	0.00
DNA transposons			25,326	14,484,075	2.04
	hobo-Activator		6,684	3,892,761	0.55
	Tourist/Harbinger		1,839	1,539,069	0.22
Rolling-circles			14,138	5,889,064	0.83
Unclassified			676,899	223,831,938	31.53
Total interspersed repeats				350,054,730	49.31
Small RNA			5,138	2,342,781	0.33
Satellites			168	53,950	0.01
Simple repeats			135,996	6,324,190	0.89
Low complexity			20,702	1,026,284	0.14

Supplementary Table 6. Transcriptome assembly statistics of *S. cumini* species

Counts of transcripts	
Total trinity 'genes'	95,459
Total trinity transcripts	204,525
GC%	43.91
**Statistics based on all transcript contigs**	
Contig N50	2,313
Median contig length (bases)	673
Average contig (bases)	1,260.52
Total assembled bases	257,807,839
**Stats based on only longest isoform per 'gene'**	
Contig N50	1,846
Median contig length (bases)	400
Average contig (bases)	866.66
Total assembled bases	82,730,644

Supplementary Table 7. KEGG pathways assigned to the coding genes of *S. cumini* species (Pathways with ≥25 genes are mentioned below)

KEGG pathways	No. of genes
Ribosome	118
Spliceosome	97
Protein processing in endoplasmic reticulum	77
Nucleocytoplasmic transport	70
Cell cycle	66
Oxidative phosphorylation	66
Ubiquitin mediated proteolysis	57
Endocytosis	55
Ribosome biogenesis in eukaryotes	55
mRNA surveillance pathway	51
Nucleotide excision repair	50
RNA degradation	49
Purine metabolism	48
Cysteine and methionine metabolism	44
MAPK signalling pathway - plant	40
Glycerophospholipid metabolism	40
Plant hormone signal transduction	39
Amino sugar and nucleotide sugar metabolism	39
Peroxisome	37
Lysosome	36
Proteasome	35
Glycolysis / Gluconeogenesis	34
Pyruvate metabolism	34
Homologous recombination	34
Glycine, serine and threonine metabolism	33
N-Glycan biosynthesis	33
Porphyrin metabolism	33
RNA polymerase	32
Terpenoid backbone biosynthesis	31

Starch and sucrose metabolism	31
DNA replication	31
Base excision repair	31
Glycerolipid metabolism	30
Pyrimidine metabolism	30
Plant-pathogen interaction	29
Glyoxylate and dicarboxylate metabolism	29
Alanine, aspartate and glutamate metabolism	28
Phagosome	28
Inositol phosphate metabolism	27
Basal transcription factors	27
Aminoacyl-tRNA biosynthesis	27
Photosynthesis	26
mTOR signalling pathway	26
Various types of N-glycan biosynthesis	25
Cellular senescence	25

Supplementary Table 8. COG categories assigned to the coding genes of *S. cumini* species

**COG categories**	**No. of genes**
Function unknown	13,259
Signal transduction mechanisms	4,623
Transcription	3,751
Posttranslational modification, protein turnover, chaperones	3,726
Carbohydrate transport and metabolism	2,905
Secondary metabolites biosynthesis, transport and catabolism	2,421
Replication, recombination and repair	1,695
Intracellular trafficking, secretion, and vesicular transport	1,657
Translation, ribosomal structure and biogenesis	1,645
Amino acid transport and metabolism	1,491
Energy production and conversion	1,488
RNA processing and modification	1,413
Lipid transport and metabolism	1,394
Inorganic ion transport and metabolism	1,276
Cell cycle control, cell division, chromosome partitioning	672
Cell wall/membrane/envelope biogenesis	595
Cytoskeleton	560
Coenzyme transport and metabolism	505
Defense mechanisms	480
Nucleotide transport and metabolism	421
Chromatin structure and dynamics	355
Nuclear structure	99
Extracellular structures	73
Cell motility	5

Supplementary Table 9. No. of *S. cumini* coding genes mapped against publicly available databases

Databases	No. of coding genes
NCBI-nr	56,403 (92.17%)
Swiss-Prot	38,046 (62.17%)
Pfam-A	35,733 (58.39%)
Overall	56,532 (92.38%)

Supplementary Table 10. List of disease susceptible genes (S-genes) in *S. cumini*

S. cumini gene ID	KO (Kegg Orthology) IDs with gene name	Target sequence ID from the DSP database (Kaur et al., 2023)
maker-scaffold462-augustus-gene-2.79-mRNA-1	K07955 (ADP-ribosylation factor-like protein 8)	522_ARL8
maker-scaffold765-augustus-gene-0.8-mRNA-1	K07374 (Tubulin alpha)	327_TOR2
maker-scaffold3418-augustus-gene-0.56-mRNA-1	K11838 (Ubiquitin carboxyl-terminal hydrolase 7)	182_AtUBP13
maker-scaffold2943-augustus-gene-0.86-mRNA-1	K11838 (Ubiquitin carboxyl-terminal hydrolase 7)	182_AtUBP13
augustus-scaffold104-processed-gene-1.6-mRNA-1	K03231 (Elongation factor 1-alpha)	543_eEF1A
augustus-scaffold104-processed-gene-2.89-mRNA-1	K03231 (Elongation factor 1-alpha)	543_eEF1A
maker-scaffold104-augustus-gene-2.117-mRNA-1	K03231 (Elongation factor 1-alpha)	543_eEF1A

Supplementary Table 11. Inter-species collinearity between *Syzygium* species

	No. of collinear blocks	No. of species-specific syntelogs	No. of total collinear genes
S. cumini* vs. *S. grande	1,792	*S. cumini* – 24,890 (40.67%)<br>*S. grande* – 20,425 (51.19%)	45,315 (44.82%)
S. aromaticum* vs. *S. grande	469	*S. aromaticum* – 18,877 (68.57%)<br>*S. grande* – 19,003 (47.62%)	37,880 (56.18%)
S. cumini* vs. *S. aromaticum	1,541	*S. cumini* – 20,919 (34.18%)<br>*S. aromaticum* – 17,594 (63.91%)	38,513 (43.41%)

Supplementary Table 12. KEGG pathways of the *S. cumini* genes present in the inter-species collinear blocks constructed with both *S. aromaticum* and *S. grande* (Pathways with ≥25 genes are mentioned)

KEGG pathway	No. of genes
Ribosome	109
Spliceosome	88
Protein processing in endoplasmic reticulum	72
Nucleocytoplasmic transport	66
Cell cycle	54
Endocytosis	49
Oxidative phosphorylation	48

Ubiquitin mediated proteolysis	47
mRNA surveillance pathway	47
Ribosome biogenesis in eukaryotes	46
RNA degradation	44
Cysteine and methionine metabolism	41
Nucleotide excision repair	41
Purine metabolism	40
Plant hormone signal transduction	39
MAPK signalling pathway - plant	38
Glycerophospholipid metabolism	37
Amino sugar and nucleotide sugar metabolism	36
Glycolysis / Gluconeogenesis	33
Homologous recombination	33
Lysosome	33
Peroxisome	33
Pyruvate metabolism	32
Starch and sucrose metabolism	31
Proteasome	31
Glycine, serine and threonine metabolism	30
N-Glycan biosynthesis	30
Glyoxylate and dicarboxylate metabolism	28
Glycerolipid metabolism	27
Pyrimidine metabolism	27
Porphyrin metabolism	27
Terpenoid backbone biosynthesis	27
Base excision repair	27
Plant-pathogen interaction	27
DNA replication	26
Alanine, aspartate and glutamate metabolism	25
Basal transcription factors	25
Aminoacyl-tRNA biosynthesis	25
Phagosome	25

Supplementary Table 13. KEGG pathways of the genes included in the species-specific gene clusters of *S. cumini* (Pathways with ≥10 genes are mentioned)

KEGG pathway	No. of genes
Ribosome	71
Protein processing in endoplasmic reticulum	44
Spliceosome	34
Nucleocytoplasmic transport	31
Amino sugar and nucleotide sugar metabolism	27
Plant hormone signal transduction	27
Glycolysis / Gluconeogenesis	26
Ubiquitin mediated proteolysis	26
Starch and sucrose metabolism	24
mRNA surveillance pathway	24
Oxidative phosphorylation	23
Cysteine and methionine metabolism	23

Peroxisome	23
Ribosome biogenesis in eukaryotes	21
RNA degradation	21
Endocytosis	21
Cell cycle	21
Pyruvate metabolism	20
Lysosome	20
Glycerophospholipid metabolism	19
Plant-pathogen interaction	19
Purine metabolism	18
Nucleotide excision repair	17
MAPK signalling pathway - plant	17
PI3K-Akt signalling pathway	17
Glyoxylate and dicarboxylate metabolism	16
Inositol phosphate metabolism	16
Terpenoid backbone biosynthesis	16
mTOR signalling pathway	16
Phagosome	16
Pentose phosphate pathway	15
Carbon fixation in photosynthetic organisms	15
Glycerolipid metabolism	15
Glycine, serine and threonine metabolism	15
Porphyrin metabolism	15
RNA polymerase	15
AMPK signalling pathway	15
Aminoacyl-tRNA biosynthesis	14
Proteasome	14
Pyrimidine metabolism	13
Arginine and proline metabolism	13
Glutathione metabolism	13
N-Glycan biosynthesis	13
Phenylpropanoid biosynthesis	13
Basal transcription factors	13
Phosphatidylinositol signalling system	13
Ascorbate and aldarate metabolism	12
Various types of N-glycan biosynthesis	12
Flavonoid biosynthesis	11
Pentose and glucuronate interconversions	11
Fructose and mannose metabolism	11
Galactose metabolism	11
Methane metabolism	11
Fatty acid biosynthesis	11
Fatty acid degradation	11
Sphingolipid metabolism	11
Valine, leucine and isoleucine degradation	11
Tryptophan metabolism	11
Phenylalanine, tyrosine and tryptophan biosynthesis	11
Ubiquinone and other terpenoid-quinone biosynthesis	10
Citrate cycle (TCA cycle)	10
Alanine, aspartate and glutamate metabolism	10

Tyrosine metabolism	10
Phenylalanine metabolism	10
Pantothenate and CoA biosynthesis	10
Wnt signalling pathway	10
Sphingolipid signalling pathway	10
Cellular senescence	10
NOD-like receptor signalling pathway	10

Supplementary Table 14. Highly expanded (>25 expanded genes) annotated gene families

Multidrug resistance protein - MATE family	'GDSL' lipolytic enzyme family	Feruloyl-CoA 6-hydroxylase (F6H)
Methyltransferase	Vacuolar iron transporter homolog	Peroxidase (PER)
Shikimate O-hydroxycinnamoyltransferase (HCT)	Protein kinase	Xyloglucan:xyloglucosyl transferase
Cinnamoyl-CoA reductase (CCR)	Glutamine synthetase	Cellulose synthase
Protein trichome birefringence-like	Flavonol synthase (FLS)	Polygalacturonase
Ras-related protein	Monoacylglycerol lipase	MFS transporter, PHS family, inorganic phosphate transporter
ABC Transporter	Cytochrome P450 family	Caffeic acid 3-O-methyltransferase (COMT)
Prolyl oligopeptidase family	Cinnamyl-alcohol dehydrogenase (CAD)	AAA ATPase family
MADS-box transcription factor	Lipolytic acyl hydrolase (LAH)	Acetylajmaline esterase (AAE)
ATP-dependent RNA helicase	E3 ubiquitin-protein ligase	Neomenthol dehydrogenase

Supplementary Table 15. KEGG pathways of the *S. cumini* genes included in the highly expanded gene families

**KEGG pathway**	**No. of genes**
Phenylpropanoid biosynthesis	5
Flavonoid biosynthesis	5
Endocytosis	5
Spliceosome	4
Pentose and glucuronate interconversions	2
ABC transporters	2
AMPK signalling pathway	2
Necroptosis	2

Supplementary Table 16. KEGG pathways of the positively selected genes in *S. cumini* (Pathways with >5 genes are mentioned)

**KEGG pathway**	**No. of genes**
Ribosome	17
Glycolysis / Gluconeogenesis	16
RNA degradation	16
Ribosome biogenesis in eukaryotes	15
Cell cycle	15
Starch and sucrose metabolism	14
Purine metabolism	14
Nucleocytoplasmic transport	14
Protein processing in endoplasmic reticulum	14
Pyruvate metabolism	13
Oxidative phosphorylation	12
Spliceosome	12
Ubiquitin mediated proteolysis	11
Proteasome	11
Endocytosis	11
Citrate cycle (TCA cycle)	10
Glyoxylate and dicarboxylate metabolism	10
Cysteine and methionine metabolism	10
Aminoacyl-tRNA biosynthesis	10
Lysosome	10
Amino sugar and nucleotide sugar metabolism	9
Glycerophospholipid metabolism	9
Phagosome	9
Synaptic vesicle cycle	9
Valine, leucine and isoleucine degradation	8
mRNA surveillance pathway	8
Plant hormone signal transduction	8
Peroxisome	8
Propanoate metabolism	7
Inositol phosphate metabolism	7
Carbon fixation in photosynthetic organisms	7
Pyrimidine metabolism	7
Ubiquinone and other terpenoid-quinone biosynthesis	7
Terpenoid backbone biosynthesis	7
Phenylpropanoid biosynthesis	7
Drug metabolism - other enzymes	7
AMPK signalling pathway	7
Cellular senescence	7
Pentose and glucuronate interconversions	6
Sulfur metabolism	6
Steroid biosynthesis	6
Glycine, serine and threonine metabolism	6
Tyrosine metabolism	6
Basal transcription factors	6
Nucleotide excision repair	6
MAPK signalling pathway – plant	6

Regulation of actin cytoskeleton	6
Plant-pathogen interaction	6

Supplementary Table 17. KEGG pathways of the *S. cumini* genes showing unique amino acid substitution with functional impact (Pathways with ≥5 genes are mentioned)

KEGG pathway	No. of genes
Protein processing in endoplasmic reticulum	13
Cell cycle	12
Starch and sucrose metabolism	11
Plant hormone signal transduction	11
Glycerophospholipid metabolism	10
MAPK signalling pathway – plant	10
Amino sugar and nucleotide sugar metabolism	9
Peroxisome	9
Spliceosome	8
Terpenoid backbone and ubiquinone and other terpenoid-quinone biosynthesis	7
Glyoxylate and dicarboxylate metabolism	7
Cysteine and methionine metabolism	7
Ribosome	7
Aminoacyl-tRNA biosynthesis	7
Wnt signalling pathway	7
Phosphatidylinositol signalling system	7
Lysosome	7
Cellular senescence	7
Plant-pathogen interaction	7
Glycolysis / Gluconeogenesis	6
Pyruvate metabolism	6
Phenylalanine, tyrosine and tryptophan biosynthesis	6
Phenylpropanoid biosynthesis	6
mRNA surveillance pathway	6
Ubiquitin mediated proteolysis	6
Proteasome	6
DNA replication	6
PI3K-Akt signalling pathway	6
Endocytosis	6
Regulation of actin cytoskeleton	6
NOD-like receptor signalling pathway	6
Flavonoid biosynthesis	5
Fructose and mannose metabolism	5
Inositol phosphate metabolism	5
Carbon fixation in photosynthetic organisms	5
Fatty acid degradation	5
Glycerolipid metabolism	5
Purine metabolism	5
Pyrimidine metabolism	5
Arginine and proline metabolism	5
Biosynthesis of various plant secondary metabolites	5

Ribosome biogenesis in eukaryotes	5
RNA degradation	5
TGF-beta signalling pathway	5
cGMP-PKG signalling pathway	5

Supplementary Table 18. KEGG pathways of the *S. cumini* genes showing higher nucleotide divergence (Pathways with >1 genes are mentioned)

KEGG pathway	No. of genes
Spliceosome	7
Ribosome	3
Oxidative phosphorylation	3
Phagosome	3
Synaptic vesicle cycle	3
Pyrimidine metabolism	2
Flavonoid biosynthesis	2
Drug metabolism - other enzymes	2
Nucleocytoplasmic transport	2
Protein processing in endoplasmic reticulum	2
Ubiquitin mediated proteolysis	2
RNA degradation	2
Lysosome	2
Circadian rhythm - plant	2

Supplementary Table 19. Gene Ontology (GO) categories that were over-represented in *S. cumini* MSA genes (TPM > 1) (Top 10 categories are mentioned below)

**Biological processes**		
**GO term ID**	**Description**	**p-value**
GO:0015748	Organophosphate ester transport	0.0073344
GO:0006414	Translational elongation	0.014264
GO:0034248	Regulation of cellular amide metabolic process	0.020711
GO:0010876	Lipid localization	0.027625
GO:0044093	Positive regulation of molecular function	0.028080
GO:0016192	Vesicle-mediated transport	0.029656
GO:0043900	Regulation of multi-organism process	0.030534
GO:0033365	Protein localization to organelle	0.036683
GO:0040011	Locomotion	0.038810
GO:0006605	Protein targeting	0.041498
**Cellular component**		
GO:0030135	Coated vesicle	0.013460
GO:0005802	Trans-Golgi network	0.017526
GO:0005798	Golgi-associated vesicle	0.027701
GO:0005730	Nucleolus	0.041986
GO:0005795	Golgi stack	0.055409
GO:0030133	Transport vesicle	0.064633
GO:0048475	Coated membrane	0.080370
GO:0030014	CCR4-NOT complex	0.16002
GO:0012506	Vesicle membrane	0.16952

GO:1904949	ATPase complex	0.17169
**Molecular function**		
GO:0005319	Lipid transporter activity	0.0099818
GO:0042578	Phosphoric ester hydrolase activity	0.015297
GO:0030276	Clathrin binding	0.025551
GO:0016741	Transferase activity, transferring one-carbon groups	0.042402
GO:0004386	Helicase activity	0.048823
GO:0001871	Pattern binding	0.053585
GO:0019899	Enzyme binding	0.086583
GO:0008237	Metallopeptidase activity	0.10679
GO:0019239	Deaminase activity	0.12891
GO:0000049	tRNA binding	0.14593

Supplementary Table 20. Gene family expansion/contraction of the enzymes involved in key secondary metabolism pathways shown in this study

Name of the enzyme	Gene family evolution
Shikimate pathway	
DAHPS	Expanded (+2)
DHQD	Expanded (+9)
SDH	Expanded (+9)
SK	Expanded (+3)
EPSPS	Expanded (+1)
CS	Expanded (+2)
Phenylpropanoid biosynthesis	
PAL	Expanded (+5)
C4H	Expanded (+2)
4CL	Expanded (+10)
HCT	Highly expanded (+37)
C3'H	Expanded (+16)
CCoAOMT	Contracted (-1)
COMT	Highly expanded (+26)
CCR	Highly expanded (+34)
CAD	Highly expanded (+32)
PER	Highly expanded (+27)
Flavonoid and anthocyanin biosynthesis	
CHS	Contracted (-1)
CHI	Expanded (+2)
F3H	Expanded (+3)
F3'H	Expanded (+16)
FLS	Highly expanded (+27)
DFR	Highly expanded (+34)
ANS	Highly expanded (+27)
Terpenoid biosynthesis	
AACT	Expanded (+2)
HMGR	Expanded (+9)
MK	Expanded (+4)
MDD	Expanded (+2)
GPPS	Expanded (+1)

GGPPS	Expanded (+4)
FOLK	Expanded (+9)
Neomenthol dehydrogenase	Highly expanded (+30)
menA	Expanded (+3)
HST	Expanded (+9)
crtB	Expanded (+2)
FDFT1	Expanded (+7)
SQLE	Expanded (+2)
Benzylisoquinoline alkaloid biosynthesis	
TAT	Expanded (+5)
TYDC	Expanded (+11)
NCS	Expanded (+2)
6OMT	Expanded (+4)
CNMT	Expanded (+1)
4OMT	Expanded (+4)
BBE	Expanded (+20)
SOMT	Expanded (+4)
CAS	Expanded (+15)
STOX	Expanded (+20)
CoOMT	Expanded (+4)

Supplementary Table 21. KEGG pathways of the *S. cumini* genes identified in the biosynthetic gene clusters (BGCs) (Pathways with >2 genes and other secondary metabolism pathways are mentioned)

KEGG pathway	No. of genes
Phenylpropanoid biosynthesis	5
Cell cycle	5
Amino sugar and nucleotide sugar metabolism	4
Plant hormone signal transduction	4
Ubiquinone and other terpenoid-quinone biosynthesis	3
Pyrimidine metabolism	3
Cysteine and methionine metabolism	3
Tyrosine metabolism	3
Tryptophan metabolism	3
Protein processing in endoplasmic reticulum	3
Phosphatidylinositol signaling system	3
Peroxisome	3
Cellular senescence	3
Terpenoid backbone biosynthesis	2
Sesquiterpenoid and triterpenoid biosynthesis	2
Flavonoid biosynthesis	2
Steroid biosynthesis	2
Monoterpenoid biosynthesis	1
Diterpenoid biosynthesis	1
Carotenoid biosynthesis	1
Stilbenoid, diarylheptanoid and gingerol biosynthesis	1
Isoflavonoid biosynthesis	1
Isoquinoline alkaloid biosynthesis	1
Tropane, piperidine and pyridine alkaloid biosynthesis	1

Supplementary Table 22. Evolutionary signatures of the *S. cumini* genes included in the BGCs

Gene name	Evolutionary signature(s)
Gibberellin receptor *GID1*	Unique substitution with functional impact
Nuclear pore localization protein NPL4	Unique substitution with functional impact and positive selection
Mannosylglycoprotein endo-beta-mannosidase	Positive selection
Xaa-Pro aminopeptidase	Positive selection
Lipoxygenase	Unique substitution with functional impact and positive selection
Transcription elongation factor S-II	Unique substitution with functional impact and positive selection
Inositol-1,3,4-trisphosphate 5/6-kinase	Positive selection
Callose synthase	Positive selection
Phosphatidylinositol 4-kinase B	Unique substitution with functional impact and higher nucleotide divergence
Alcohol dehydrogenase class-P	Positive selection
Jasmonic acid-amino synthetase	Positive selection
Hydroxymethylglutaryl-CoA lyase	Unique substitution with functional impact
La-related protein 7	Positive selection
Mannosyl-oligosaccharide alpha-1,2-mannosidase	Unique substitution with functional impact
Squalene monooxygenase	Unique substitution with functional impact and positive selection
Spastin	Positive selection
1,4-dihydroxy-2-naphthoate polyprenyltransferase (menA)	Unique substitution with functional impact and positive selection
Cellulose synthase A	Positive selection
UDP-glucose 4,6-dehydratase	Unique substitution with functional impact
Origin recognition complex subunit 3	Unique substitution with functional impact

Note: Genes that were assigned KO (Kegg Orthology) IDs are mentioned above

Supplementary Table 23. Key BGC genes of *S. cumini* involved in secondary metabolites biosynthesis

Gene symbol	Gene name
CAD	Cinnamyl-alcohol dehydrogenase
SQLE	Squalene monooxygenase
4CL	4-coumarate--CoA ligase
crtB	15-cis-phytoene synthase
menA	1,4-dihydroxy-2-naphthoate polyprenyltransferase
CCR	Cinnamoyl-CoA reductase
HCT	Shikimate O-hydroxycinnamoyltransferase
GGPPS	Geranylgeranyl diphosphate synthase
-	(+)-neomenthol dehydrogenase
PER	Peroxidase
POR	Protochlorophyllide reductase
AOC3	Primary-amine oxidase
LOX2S	Lipoxygenase
dgkA	Diacylglycerol kinase (ATP)
FLAD1	FAD synthetase

ispD	2-C-methyl-D-erythritol 4-phosphate cytidylyltransferase
ISA	Isoamylase
dapA	4-hydroxy-tetrahydrodipicolinate synthase
ACS	1-aminocyclopropane-1-carboxylate synthase
KAO	ent-kaurenoic acid monooxygenase
GA3ox	Gibberellin 3beta-dioxygenase
ANR	Anthocyanidin reductase
CYP710A	Sterol 22-desaturase
UGT72E	Coniferyl-alcohol glucosyltransferase
APG1	MPBQ/MSBQ methyltransferase
AFS1	Alpha-farnesene synthase
SMO1	Plant 4,4-dimethylsterol C-4alpha-methyl-monooxygenase
ADH1	Alcohol dehydrogenase class-P
CYP76A26	Nepetalactol monooxygenase

Supplementary Table 24. Exon-intron numbers of the key genes involved in phenylpropanoid-flavonoid (PF) biosynthesis and terpenoid biosynthesis pathways in the three *Syzygium* species

Gene name	*S. cumini*		*S. aromaticum*		*S. grande*	
	Exons	Introns	Exons	Introns	Exons	Introns
**Phenylpropanoid biosynthesis**						
PAL	2	1	2	1	2	1
C4H	2	1	2	1	2	1
4CL	9	8	9	8	-*	
HCT	2	1	-*		2	1
C3'H	2	1	2	1	2	1
CCoAOMT	6	5	6	5	6	5
COMT	5	4	2	1	2	1
CCR	10	9	9	8	10	9
F5H	2	1	2	1	2	1
CAD	6	5	6	5	-*	
PER	7	6	7	6	7	6
**Flavonoid and anthocyanin biosynthesis**						
CHS	9	8	9	8	9	8
CHI	2	1	2	1	2	1
F3H	4	3	4	3	3	2
F3'H	2	1	2	1	2	1
FLS	4	3	4	3	4	3
DFR	10	9	9	8	10	9
ANS	4	3	4	3	4	3
**Terpenoid biosynthesis**						
GPPS	6	5	6	5	6	5
FPPS	12	11	12	11	12	11
GGPPS	4	3	4	3	4	3
FOLK	11	10	11	10	11	10
Neomenthol dehydrogenase	10	9	-*		6	5
menA	9	8	2	1	9	8
HST	3	2	3	2	3	2

APG1	4	3	4	3	4	3
crtB	5	4	5	4	5	4

*Raw score was not suitable in Exonerate analysis

Supplementary Table 25. Length of the key genes involved in PF biosynthesis and terpenoid biosynthesis pathways in the three *Syzygium* species

Gene name	*S. cumini* (bases)	*S. aromaticum* (bases)	*S. grande* (bases)
**Phenylpropanoid biosynthesis**			
PAL	4,668	4,653	4,717
C4H	1,952	1,940	1,955
4CL	5,194	5,174	-*
HCT	2,171	-*	2,026
C3'H	1,049	1,049	1,049
CCoAOMT	2,370	2,392	2,367
COMT	2,918	623	639
CCR	5,282	5,244	5,290
F5H	1,472	1,261	1,486
CAD	4,702	4,750	-*
PER	4,171	4,525	7,821
**Flavonoid and anthocyanin biosynthesis**			
CHS	5,236	9,852	5,236
CHI	590	590	590
F3H	2,963	2,961	2,283
F3'H	1,049	1,049	1,049
FLS	2,332	2,334	2,321
DFR	5,282	5,244	5,290
ANS	2,332	2,334	2,321
**Terpenoid biosynthesis**			
GPPS	5,796	5,821	5,813
FPPS	4,577	4,582	4,591
GGPPS	2,880	2,802	2,881
FOLK	3,204	6,380	7,166
Neomenthol dehydrogenase	9,649	-*	4,019
menA	5,121	1,253	5,129
HST	2,207	2,199	2,210
APG1	3,899	3,906	3,904
crtB	2,856	2,850	2,858

*Raw score was not suitable in Exonerate analysis

SUPPLEMENTARY FIGURES

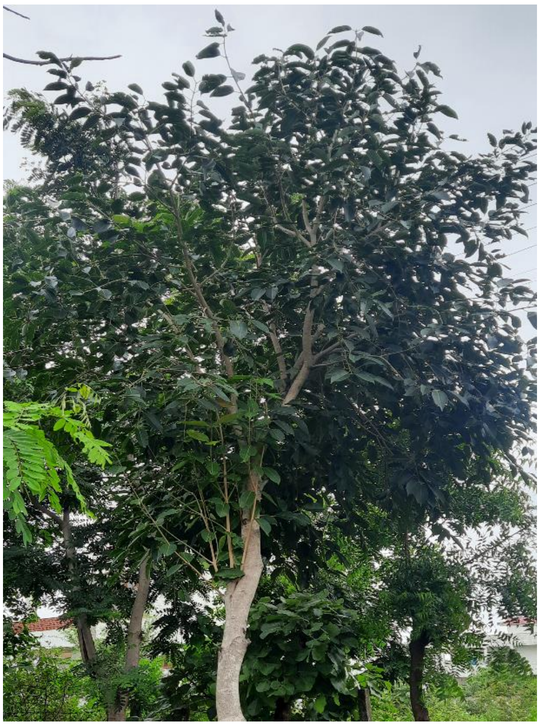

Supplementary Figure 1. *S. cumini* tree that was used for genome sequencing in this study.

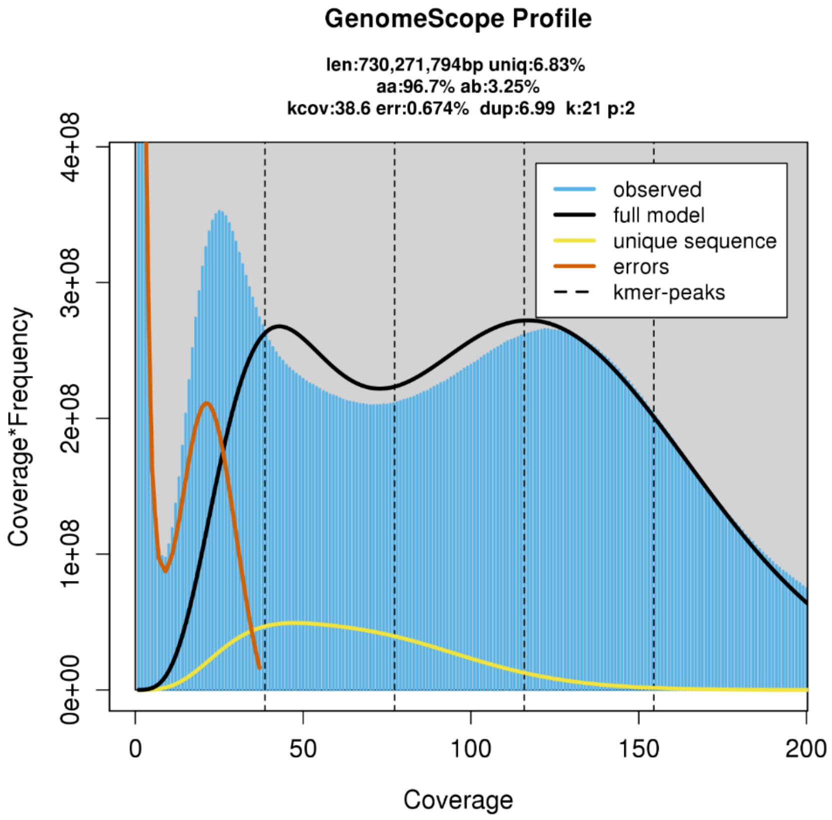

Supplementary Figure 2. GenomeScope profile of *S. cumini* genome showing genome size and heterozygosity (Ranallo-Benavidez et al., 2020).

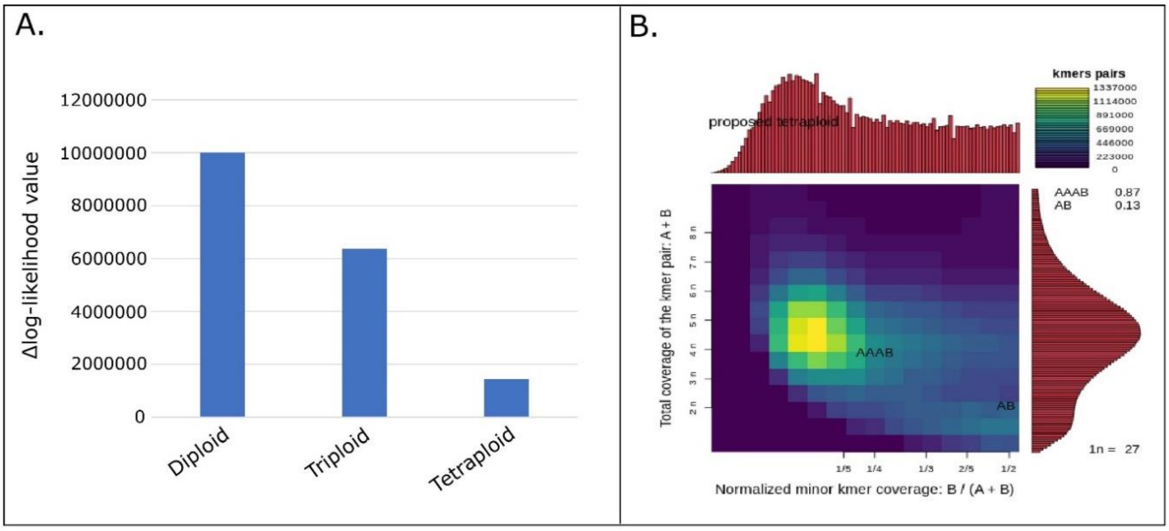

Supplementary Figure 3. Ploidy level estimation for *S. cumini* genome. **A.** Δlog-likelihood values for the three fixed models using nQuire (Weib et al., 2018), **B.** Smudgeplot profile for *S. cumini* genome (Ranallo-Benavidez et al., 2020).

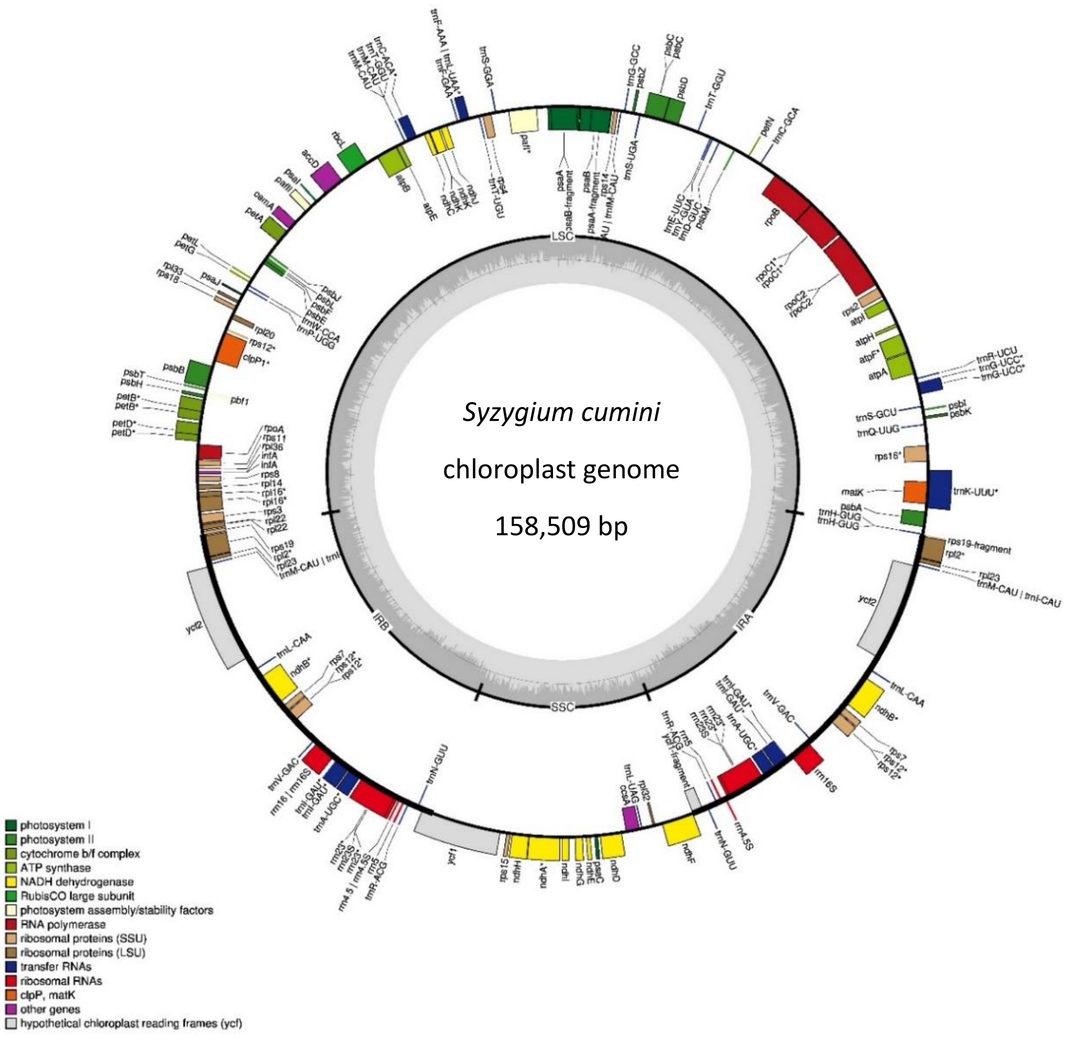

Supplementary Figure 4. Chloroplast genome annotation of *S. cumini*.

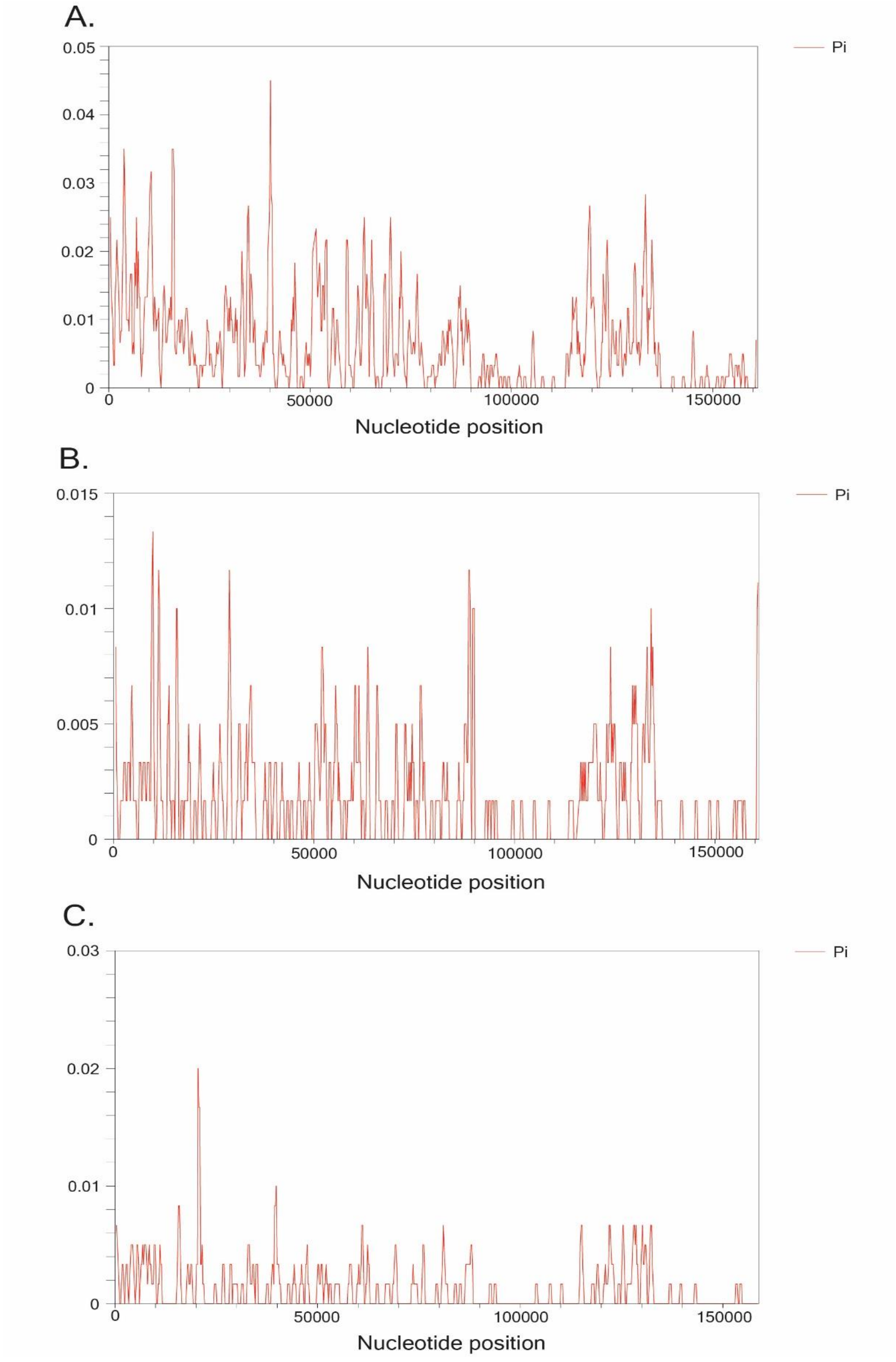

Supplementary Figure 5. Nucleotide diversity (π) across the *S. cumini* chloroplast genomic positions. **A.** between this study and GQ870669.3 (Asif et al., 2013), **B.** between this study and NC_053327.1, **C.** between this study and MN095412.1. Sliding window and step size were set to 600 bp and 200 bp, respectively.

SUPPLEMENTARY TEXT

DNA extraction

The leaves were collected and cleaned to extract nucleic acid (DNA/RNA). The leaves were taken immediately for RNA extraction. The washed leaves (~3 µg in weight) were homogenized in liquid nitrogen using a pre-cooled autoclaved mortar and pestle. The homogenized leaves were taken in a 50 ml centrifuge tube with 20 mL of Carlson lysis buffer. Carlson lysis buffer was pre-heated at 65˚C for 30 mins. The lysis process was supplemented with the addition of 100 µl of β-mercaptoethanol, 200 µl of Proteinase K (Qiagen, CA, USA), and 100 µl of RNase A (PureLink, ThermoFisher), and incubated at 65˚C for 1 hr with intermittent mixing by inverting the tubes in every 15 mins. After 1 hr, the tube was allowed to cool at room temperature (RT), and subsequently, 100 µl of RNase A was added and incubated at 37˚C for 30 mins for RNA degradation. After RNase treatment, the sample was purified thrice with an equal volume of chloroform: isoamyl alcohol (ratio 24:1) and centrifuged at 4,500xg for 15 mins. The final aqueous phase was collected in a new centrifuge tube and 0.7x ice-cold isopropanol was added. The tube was mixed by inverting it slowly to avoid fragmentation of DNA. The DNA precipitation was facilitated by overnight incubation at room temperature. The room temperature incubation inhibits polysaccharide precipitation along with DNA. The DNA was pelleted down by centrifuging at 5,000xg for 10 mins and washed thrice with 70% ethanol. The DNA pellet was air dried, eluted in 200 µl of nuclease-free water (NFW), and incubated at 37˚C for 10 mins. The quality of DNA was checked on 0.8% agarose gel electrophoresis as well as Nanodrop 8000 spectrophotometer. The DNA was quantified using a qubit ds DNA BR assay kit on Qubit 2.0 fluorometer (Life Technologies, United States).

Species identification assay

The extracted DNA was used to amplify two DNA markers: *ITS2* (Internal Transcribed Spacer) and *MatK* (Maturase K). The amplification was performed on Veriti 96 well thermal cycler (Applied Biosystems) using the following primers:

1. *ITS2* forward primer: 5′-GCATCGATGAAGAACGCAGC-3′
   ITS2 reverse primer: 5′-TCCTCCGCTTATTGATATGC-3′
2. *MatK* forward primer: 5′-CGATCTATTCATTCAATATTTC-3′
   MatK reverse primer: 5′-TCTAGCACACGAAAGTCGAAGT-3′

The amplification was evaluated on 2% agarose gel, followed by the purification of amplicons using a PureLink PCR purification kit (Invitrogen, USA). The purified amplicons were sequenced on a Sanger sequencer. The obtained amplicon sequences were aligned against NCBI non-redundant nucleotide database (nt) using BLASTN.

Genomic sequencing

The extracted DNA was used to prepare the library on the Chromium controller instrument using Chromium Genome Library and Gel Bead Kit (10x Genomics). The 10x Genomics library was sequenced on Illumina NovaSeq 6000 instrument for generating 150 bp paired-end reads. The DNA was purified using Ampure XP magnetic beads (Beckman Coulter, USA) for Nanopore sequencing. The purified DNA was used to prepare the Nanopore library using SQK-LSK109 and SQK-LSK110 library preparation kit (Oxford Nanopore Technologies, UK). The library was loaded on flowcell and sequenced on a MinION Mk1C sequencer.

RNA extraction and sequencing

The RNA was extracted following a similar method that was used for *Syzygium longifolium species* with a few modifications (Soewarto et al., 2019). The RNA precipitation was performed directly using two volumes of 100% ethanol and 1/10 volume of 3M sodium acetate and incubated at 4˚C overnight. The RNA was washed and purified using a RNeasy mini kit (Qiagen, CA, USA). The RNA quality was diluted ten times and was quantified on Qubit 2.0 fluorometer using a qubit ss RNA HS kit (Life Technologies, United States). Quality of the RNA was evaluated using High Sensitivity D1000 ScreenTape on Agilent 2200 TapeStation (Agilent, Santa Clara, CA). The RNA library was prepared using TruSeq Stranded Total RNA Library Preparation kit with the Ribo-Zero Plant workflow (Illumina Inc., CA, USA). The transcriptome library was sequenced to generate 150 bp paired-end reads on Illumina NovaSeq 6000 instrument.

Gene structure analysis results

Gene structure analysis of the key genes involved in the PF biosynthesis pathway and terpenoid pathway of secondary metabolism (**Figures 4-5**) showed the presence of a similar number of exons for the corresponding genes in all three *Syzygium* species - *S. cumini*, *S. aromaticum*, and *S. grande* (**Supplementary Table 24)**. The gene lengths were also similar in the three *Syzygium* species except for *COMT*, *PER*, *CHS*, *F3H*, *FOLK*, *menA*, and neomenthol dehydrogenase due to an increase in intron numbers and intron length (**Supplementary Table 25**).

REFERENCES

Asif, H., Khan, A., Iqbal, A., Khan, I. A., Heinze, B., and Azim, M. K. (2013). The chloroplast genome sequence of Syzygium cumini (L.) and its relationship with other angiosperms. *Tree Genet. Genomes* 9, 867–877. doi: 10.1007/S11295-013-0604-1/FIGURES/6.

Kaur, S., Bishnoi, R., Priyadarshini, P., Singla, D., and Chhuneja, P. (2023). DSP: database of disease susceptibility genes in plants. *Funct. Integr. Genomics* 23, 1–4. doi: 10.1007/S10142-023-01132-X/FIGURES/1.

Ranallo-Benavidez, T. R., Jaron, K. S., and Schatz, M. C. (2020). GenomeScope 2.0 and Smudgeplot for reference-free profiling of polyploid genomes. *Nat. Commun.* doi: 10.1038/s41467-020-14998-3.

Soewarto, J., Hamelin, C., Bocs, S., Mournet, P., Vignes, H., Berger, A., et al. (2019). Transcriptome data from three endemic Myrtaceae species from New Caledonia displaying contrasting responses to myrtle rust (Austropuccinia psidii). *Data Br.* 22, 794. doi: 10.1016/J.DIB.2018.12.080.

Tao, L., Shi, Z. G., and Long, Q. Y. (2020). Complete chloroplast genome sequence and phylogenetic analysis of Syzygium malaccense. *http://www.tandfonline.com/action/authorSubmission?journalCode=tmdn20&page=instructions* 5, 3567–3568. doi: 10.1080/23802359.2020.1829132.

Weib, C. L., Pais, M., Cano, L. M., Kamoun, S., and Burbano, H. A. (2018). nQuire: A statistical framework for ploidy estimation using next generation sequencing. *BMC Bioinformatics*. doi: 10.1186/s12859-018-2128-z.
